# Improving the Efficiency of Vγ9Vδ2 T-Cell Immunotherapy in Cancer

**DOI:** 10.3389/fimmu.2018.00800

**Published:** 2018-04-19

**Authors:** Timm Hoeres, Manfred Smetak, Dominik Pretscher, Martin Wilhelm

**Affiliations:** Department of Hematology and Medical Oncology, Paracelsus Medical University, Nuremberg, Germany

**Keywords:** gamma delta T-cell, cancer immunotherapy, tumor metabolism, ADCC, NKG2D, immune checkpoints, programmed cell death protein 1, vascular endothelial growth factor

## Abstract

Increasing immunological knowledge and advances in techniques lay the ground for more efficient and broader application of immunotherapies. gamma delta (γδ) T-cells possess multiple favorable anti-tumor characteristics, making them promising candidates to be used in cellular and combination therapies of cancer. They recognize malignant cells, infiltrate tumors, and depict strong cytotoxic and pro-inflammatory activity. Here, we focus on human Vγ9Vδ2 T-cells, the most abundant γδ T-cell subpopulation in the blood, which are able to inhibit cancer progression in various models *in vitro* and *in vivo*. For therapeutic use they can be cultured and manipulated *ex vivo* and in the following adoptively transferred to patients, as well as directly stimulated to propagate *in vivo*. In clinical studies, Vγ9Vδ2 T-cells repeatedly demonstrated a low toxicity profile but hitherto only the modest therapeutic efficacy. This review provides a comprehensive summary of established and newer strategies for the enhancement of Vγ9Vδ2 T-cell anti-tumor functions. We discuss data of studies exploring methods for the sensitization of malignant cells, the improvement of recognition mechanisms and cytotoxic activity of Vγ9Vδ2 T-cells. Main aspects are the tumor cell metabolism, antibody-dependent cell-mediated cytotoxicity, antibody constructs, as well as activating and inhibitory receptors like NKG2D and immune checkpoint molecules. Several concepts show promising results *in vitro*, now awaiting translation to *in vivo* models and clinical studies. Given the array of research and encouraging findings in this area, this review aims at optimizing future investigations, specifically targeting the unanswered questions.

## Introduction

Following the discovery in the 1980s, gamma delta (γδ) T-cells have become increasingly recognized as important players in natural host defense against infections and malignancies. Early evidence of an anti-tumor functionality of γδ T-cells came from the experiments in mice (1) and it is now well established (2). In humans, γδ T-cells can be found in various cancer tissue samples [e.g., melanoma (3, 4) and epithelial tumors (5–11)]. More recently, analysis of microarray data also described patterns of γδ T-cells in a large collection of malignancies (12) and a prior extensive gene expression study demonstrated that γδ T-cell infiltration into tumors represents a positive prognostic marker in many types of cancer (13). Offering some hints for a functional role in tumor rejection, γδ T-cell infiltration in melanoma, colorectal cancer, and lung tumors were found to be associated with lower stage and lack in metastasis. Additionally, γδ T-cells extracted from such cancer tissues were able to kill malignant cells *in vitro* (4, 14, 15). In cancer patients, γδ T-cells were also repeatedly found reduced or defective and depicted a diminished proliferative capacity (16–18) and exhaustion (19–23). Patients with higher γδ T-cell count following allogenic stem cell transplantation for acute leukemia had a significant survival advantage (24). In connection with their suspected function in natural tumor defense, the utilization of γδ T-cells has become a promising concept in the field of cancer immunotherapy.

### Definition

γδ T-cells express variables Vγ and Vδ chains (25, 26) as part of a T-cell receptor (TCR) complex that is structurally and functionally distinctive from the major histocompatibility complex (MHC) binding TCR of αβ T-cells (27). In humans, it is feasible to further divide γδ T-cells into “Vδ2” and “non-Vδ2 cells,” the latter consisting of mostly Vδ1- and rarely Vδ3- or Vδ5-chain expressing cells. Despite unrestricted and the theoretically high combinatory diversity (28), the Vδ2 chain is found preferentially paired with the Vγ9 chain (29). These Vγ9Vδ2 T-cells account for approximately 5% of peripheral blood T-cells, representing the dominant γδ T-cell subpopulation in this compartment in healthy human adults (30). Interestingly, the preferential appearance of Vγ9- and Vδ2-chains develops in the fetus (31), but the overall clonal repertoire of blood γδ T-cells is further contracting after birth (32). The latter is probably a response to a uniform stimulus, like a ubiquitous pathogen or conserved stress molecule (33).

### Functional Aspects

Genetic and functional studies indicate that γδ T-cells have developed and act as an intermediate between the innate and the adaptive immune system. Features representative of an innate phenotype is their ability to mediate antibody-dependent cell-mediated cytotoxicity (ADCC) and phagocytosis and to rapidly react toward pathogen-specific antigens without prior differentiation or expansion (28). Notably, the gene expression signature of Vγ9Vδ2 T-cells was characterized as a hybrid of αβ and NK-cells (34). Typical characteristics of the adaptive immune system, found in γδ T-cells, are their capabilities for somatic recombination of receptor genes, memory formation (35), and professional antigen presentation (36). Unlike αβ T-cells, γδ T-cells respond directly to proteins and non-peptide antigens (37) and are therefore not MHC restricted (38). At least some γδ T-cell specific antigens display evolutionary conserved molecular patterns, found in microbial pathogens and “induced self-antigens,” which become upregulated by cellular stress, infections, and transformation (28). Following the observation on stimulatory effects of certain non-peptide mycobacterial components on Vγ9Vδ2 T-cells (39, 40), the responsible substances could be isolated and characterized and are commonly termed as phosphoantigens (PAgs) (41). We consider PAgs the primary trigger of Vγ9Vδ2 γδ T-cell activation and discuss them in greater detail in the following. However, Vγ9Vδ2 γδ T-cells may also respond to other antigens and ligands *via* TCR and (co-)receptors (42).

### Vγ9Vδ2 T-Cells in Cancer Immunotherapy

Subsets of Vγ9Vδ2 T-cells can be defined analyzing the expression of surface markers (e.g., CD27, CD45RA, CCR7, and CD16) or regarding their dominant cytokine production and correlate with functional differences like proliferative capacity or cytotoxic potential (43, 44). It has been extensively demonstrated *in vitro* (45–55) and using *in vivo* models (22, 56–68) that γδ T-cells are able to recognize various tumor cells and exert strong anti-tumor effects. Tumor growth is inhibited *via* different mechanisms including the release of pro-inflammatory cytokines, granzymes and perforin, and the engagement of apoptosis inducing receptors (69).

Several drugs and treatment concepts might improve the activity of Vγ9Vδ2 T-cells against cancer. Most candidates are still at a pre-clinical stage, some were tested in animal models, and very few went into clinical tests so far. Although Vδ1+ cells shown promising results pre clinically (70), all previous clinical trials focused on the usage of Vγ9Vδ2 T-cells. Reasons for the earlier therapeutic employment of Vγ9Vδ2 T-cells include their relatively high abundance in the peripheral blood and the possibility to efficiently culture them *ex vivo* or to stimulate and expand them *in vivo* using amino-bisphosphonates (N-BP) or synthetic PAgs (45), as discussed later.

Here, we divide the existing clinical studies according to the used strategy into two main groups: (1) *in vivo* activation (17, 18, 23, 71–74) and (2) adoptive cell transfer strategies (75–84). In the latter case, the adoptively transferred cells originally were extracted, activated, and cultured autologous blood cells. Varieties include the transfer of processed haploidentical cell preparations with subsequent *in vivo* stimulation (82), as well as local administration of cultured cells into the tumor or the peritoneal cavity (85, 86). Well organized and comprehensive analyses of the performed clinical studies involving γδ T-cells have recently been published by others (45, 87, 88) and an overview is given in Table 1.

**Table 1:** Clinical studies.

Reference	Year	Disease	*N*	Reported outcome	Systemic therapy/comments
***In vivo* stimulation**					
Wilhelm et al. (18)	2003	MM, indolent, lymphomas	19	16% PR, 16% SD	+PAM +IL-2/response correlates with *in vitro* expansion
Dieli et al. (23)	2007	HRPC	18	16% PR, 27% SD	+ZOL +IL-2
Bennouna et al. (73)	2010	RCC, GYN-, GI-cancers	28	42% SD	+BrHPP +IL-2
Laurent et al. (89) abstract only	2010	Follicular lymhoma	45	26% CR, 18% PR	+BrHPP +IL-2 +RTX
Meraviglia et al. (71)	2010	Breast cancer	10	10% PR, 20% SD	+ZOL +IL-2/response correlates with *in vivo* expansion
Lang et al. (74)	2011	RCC	12	16% SD	+ZOL +IL-2
Kunzmann et al. (72)	2012	RCC, melanoma, AML	21	16–42% SD	+ZOL +IL-2
				AML: 25% PR	
Pressey et al. (17)	2016	Neuroblastoma	4	25% SD, 75% PD	+ZOL +IL-2

**Adoptive transfer**					
Kobayashi et al. (78)	2007	RCC	7	Delayed tumor doubling times in 4/7 patients	–
Bennouna et al. (75)	2008	RCC	10	60% SD	–
Abe et al. (80)	2009	MM	6	66% SD	–
Nakajima et al. (81)	2010	Lung cancer	10	30% SD	–
Kobayashi (79)	2011	RCC	11	9% CR, 45% SD	+ZOL +IL-2
Nicol et al. (84)	2011	Solid tumors	18	16% SD, 16% PR and CR	+ZOL +other tumor-specific treatments
Noguchi et al. (77)	2011	Solid tumors	25	12% SD, 12% PR	+other tumor-specific treatments
Sakamoto et al. (76)	2011	Lung cancer	15	40% SD	–
Cui et al. (86)	2014	HCC	62	Longer PFS and OS	–/in addition to radiofrequency ablation
Wilhelm et al. (82)	2014	Hematological malignancies	4	75% CR	+ZOL +IL-2 +Chemo/*in vivo* stimulation following transfer of haploidentical cells
Wada et al. (85)	2014	Gastric cancer	7	Reduction in ascites in 2/7 patients	–/intraperitoneal administration of γδ T-cells
Aoki et al. (90)	2017	Pancreatic cancer—adjuvant	28	Higher recurrence free survival in patients with sustained higher γδ T-cell numbers	+Chemo

*AML, acute myeloid leukemia; BrHPP, bromohydrin pyrophosphate; Chemo, chemotherapy; CR, complete remission, GI, gastrointestinal; GYN, gynecological; HCC, hepatocellular carcinoma; HRPC, hormone refractory prostate cancer; MM, multiple myeloma; N, number of patients; OS, overall survival; PAM, pamidronic acid; PD, progressive disease; PFS, progression free survival; PR, partial remission; RCC, renal cell carcinoma; RTX, rituximab; SD, stable disease; ZOL, zoledronic acid*.

### Outline

Much has been learned by studying γδ T-cells from animals, especially those from mice. However, there are major distributional, structural, and functional differences between the species, especially the lack of Vγ9Vδ2 T-cells or functional homologs in mice (91, 92). In this review, we focus on human γδ T-cells, their anti-tumor capabilities, and strategies for improving the effectiveness of Vγ9Vδ2 T-cells in cancer immunotherapy. Current publications contain additional information on the topics not covered here, especially the biology of non-Vδ2 cells (93) and their role in cancer and cancer therapy (2). We also refer to more detailed literature regarding the differences of rodent and human γδ T-cells (28), γδ T-cells acting as professional antigen-presenting cells (36), concerning B-cell help (94) and potential use as a vaccine (95), cell ontogeny (33), phylogenetic aspects (28, 42), genetically modified γδ T-cells (e.g., CARs) (96, 97), as well as molecular details of receptor signaling (98, 99). We discuss approaches especially that aim to sensitize target cells and the local interaction of tumor and effector cells in connection with the underlying mechanisms.

## Targeting the Cellular Metabolism

Survival and growth of cancer cells are connected to specific metabolic alterations which have been considered a distinctive “hallmark of cancer” (100). Most prominent example of such adaptation is the “Warburg effect,” the preferential utilization of aerobic glycolysis by various tumor cells, described by Warburg in 1924 (101). Obvious elements of this phenotype are the inhibition of oxidative phosphorylation despite sufficient oxygenation, an elevated glucose consumption, and an increased production of lactic acid (LA). Changes in the tumor metabolism can be complex and beside glucose metabolism also affect lipid and amino acid pathways (102). Correspondingly, our idea of Vγ9Vδ2 T-cell natural anti-tumor functions is based on their ability to distinguish normal and transformed cells due to their metabolic phenotype. In particular, they might recognize an intrinsic overproduction of PAgs arising from isoprenoid biosynthesis in tumor cells.

Many PAgs are naturally occurring prenyl-pyrophosphates (41) originating from isopentenyl pyrophosphate (IPP) of the eukaryotic mevalonate pathway as well as those generated in the microbial non-mevalonate (also termed as MEP or DOX-P) pathway (103). A dysregulated mevalonate pathway, conjoined with a higher abundance of mevalonate pathway products was described in certain malignant cell types (104, 105) and may indeed be important to support the survival of malignant cells (106). PAg accumulation has been explained by increased buildup, especially of IPP due to upregulation of the gate-keeping enzyme 3-hydroxy-3-methyl-glutaryl-coenzyme A reductase (107) and other mevalonate pathway enzymes (104). We currently lack sufficient information to decide if a dysregulated mevalonate pathway associated with increased PAgs is indeed a “general hallmark of tumorigenesis” rather than an outlier. In any case, several therapeutic concepts focus on Vγ9Vδ2 T-cells’ metabolic sensor and potent effector mechanisms.

### N-BPs and PAgs

Activation of Vγ9Vδ2 T-cells with PAgs and N-BPs is the most commonly used strategy for *in vitro* research and both *in vivo* stimulation as well as application of adoptive cell therapy. The potency of the individual PAg molecule to elicit response from Vγ9Vδ2 T-cells differs (108) and is especially high for microbial (E)-4-hydroxy-3-methyl-butenyl pyrophosphate (HMBPP), certain synthetic compounds like bromohydrin pyrophosphate (BrHPP) (109) or nucleotides derived from HMBPP (110). However, so far only BrHPP and N-BPs have been used clinically. N-BPs were found to trigger activation and expansion of Vγ9Vδ2 T-cells as well as their interferon-γ (IFN-γ) release (46, 111) and were later recognized as indirect acting PAgs (112). This class of substances is structurally related to direct PAgs, but acts by inhibition of farnesyl diphosphate synthase and the accumulation of upstream metabolites like the direct PAg IPP (113). In immunotherapy N-BPs serve a double purpose. First, they sensitize target cells, rendering many primarily resistant tumor cells vulnerable to γδ T-cell mediated attack (114). Second, they induce expansion of γδ T-cells *in vivo* and *in vitro*. The degree of inhibition of farnesyl diphosphate synthase thereby correlates well with important anti-tumor functions of Vγ9Vδ2 T-cells over various tumor cell lines (115). Apart from sensitization of tumor cells, N-BPs exert additional direct anti-neoplastic effects, like an increased production of toxic mevalonate pathway products and a decrease of essential downstream metabolites (113, 116).

#### *Ex Vivo* Culture and *In Vivo* Models

Potent natural and synthetic PAgs, like the patented drug BrHPP (termed as IPH1101 or Phosphostim^®^) can be used for effective *in vitro* (117) and *in vivo* (22, 75) expansion of Vγ9Vδ2 T-cells.

Protocols for *ex vivo* culture of human Vγ9Vδ2 T-cells vary regarding the culturing conditions, timing and dosage of used N-BPs or PAgs, and added co-stimulators like IL-2 (88, 118) and may result in different phenotypes and effector cell characteristics. Zoledronic acid (ZOL) is a potent N-BP and commonly used about 1 µM *in vitro*, a concentration also in the range of the peak plasma level following a single standard dose of 4 mg intravenously (88). Repetitive administration of exogenous IL-2 is commonly used as it drives proliferation of PAg stimulated Vγ9Vδ2 T-cells resulting in an increased yield (63, 67). Results of *in vitro* expansion are highly donor dependent and may also predict the respective *in vivo* expansion efficacy, which can be additionally restricted in cancer patients (18). Currently, an optimal dose of ZOL as well IL-2 has not been determined *in vivo* (88) and a recent study indicated that the efficacy of ZOL stimulation depends on drug concentration and duration of exposure with an individual optimum (67).

The ability to recognize the PAgs is linked to germline-encoded regions of the γδ TCR (119) and so far functionally only described in primates (120). Even though homologs sequences of human Vγ9 and Vδ2 genes were recently described in other species, such as alpaca and armadillo (121, 122). As wild type mice lack PAg-responding γδ T-cells the *in vivo* expansion of human Vγ9Vδ2 T-cells has been studied using xenograft mice (57, 123) or cynomolgus monkeys (59). Results from such models show that sensitizing tumor cells with N-BPs, combined with adoptive transfer of *ex vivo* expanded human Vγ9Vδ2 T-cells with or without exogenous IL-2 administration is feasible and induces moderate anti-tumor responses (58, 65, 66, 68, 124). The role for additional systemic application of N-BPs in context with adoptive cell transfer strategies remains uncertain. On one side it has been reported to promote engraftment of *ex vivo* stimulated and adoptively transferred human cells in mice (124), on the other side there are indications that repetitive application of these drugs *in vivo* induces Vγ9Vδ2 T-cells exhaustion (23, 71, 74).

#### Clinical Experience

One may speculate that the observed anti-tumor effects of N-BPs or high-dose IL-2 monotherapy as well as allogenic stem cell transplantation are influenced by γδ T-cells without being recognized as such (125–127). Implementation of clinical Vγ9Vδ2 T-cell studies benefited from the fact that side effects and pharmacological profiles of N-BPs and IL-2 monotherapy were already known. IL-2 is established as an effective treatment for several types of cancer for about 30 years (128) and N-BPs are widely used for osteoporosis, hypercalcemia, and the treatment of bone metastasis (125). The first prospective trial focusing on the *in vivo* stimulation of anti-tumor functions by γδ T-cells used the N-BP pamidronic acid (18), later studies the more potent ZOL (17, 23, 71, 72, 74) in combination with IL-2. These N-BPs have also been used to stimulate Vγ9Vδ2 T-cells *ex vivo* for adoptive cell therapy (76, 77, 80, 81, 83). Additionally, a few studies applying adoptive cell transfer included the systemic administration of ZOL with (79, 82) or without additional IL-2 (84). Taken together the clinical studies involving the use of N-BPs to increase the anti-tumor effects of Vγ9Vδ2 T-cells in different types of malignancies depicted a tolerable toxicity but revealed inconsistent responses and overall only a modest efficacy (compare Table 1).

Similarly, BrHPP was tested in early clinical studies with small success, for both *ex vivo* stimulation and consecutive adoptive transfer of cells in combination with IL-2 in metastatic renal cell carcinoma (75) and for *in vivo* stimulation targeting solid tumors (73). A strategy combining BrHPP stimulation and the tumor targeting antibodies rituximab (RTX) (89) is discussed separately.

#### Current Obstacles

Several reasons might explain the limited therapeutic effectiveness of both N-BPs and synthetic PAgs *in vivo*. Maybe most importantly N-BPs and synthetic PAgs lack cancer specificity regarding uptake or molecular targeting and also affect other cells. Also, N-BPs and BrHPP both have short plasma half-life periods (22, 67). BrHPP is quickly degraded by plasma phosphatases and common N-BPs cannot passively cross the plasma membrane, and is preferentially rooted to the bone due to their calcium binding characteristics (112). Cancer cells in other compartments are those that lack adequate active transport mechanisms might therefore not be affected. It is established that monocytes/macrophage type cells take up N-BPs *via* fluid endocytosis and induce activation of Vγ9Vδ2 T-cells (129, 130). Unfortunately ZOL also induces killing of human macrophages (131) and, additionally, uptake of N-BP by neutrophils impairs γδ T-cell proliferation *via* production of reactive oxygen species (132). Indeed treatment with N-BP can decrease circulating γδ T-cell count (133) and repetitive stimulation with BrHPP lead to progressive exhaustion of γδ T-cell activation and expansion *in vivo* (22). A new strategy to stimulate Vγ9Vδ2 T-cells and avoid exhaustion might be the application of an attenuated, live vaccine with genetically engineered metabolic profile that overproduces HMBPP. Adapting traits of a bacterial infection with *Salmonella enterica* indeed elicited a prolonged Vγ9Vδ2 T-cell immunity in monkeys (134). A different concept to increase N-BP concentration in the tumor tissue is to administer drugs (and *ex vivo* stimulated cells) locally (135). Nevertheless, this is not a working concept for systemic diseases. It also has to be taken into account that although commonly well tolerated, N-BPs and exogenous IL-2 have considerable and dose limiting toxicities, including inflammatory and cytokine reactions, osteonecrosis of the bone, and hypocalcemia (128, 136).

#### Modified PAgs and N-BPs

The development of new direct and indirect PAgs may overcome pharmacodynamic restrictions and improve clinical efficacy (112). Newly designed PAgs (137) and bisphosphonate prodrugs (138, 139) have chemically masked phosphate groups, allowing these compounds to enter cells without the need for active transmembrane transport (140) and should not accumulate in the bone. Following intracellular uptake they are converted to their active forms, which are potent stimulators of Vγ9Vδ2 T-cells and sensitize different tumor cell lines toward γδ T-cell anti-tumor effects *in vitro* (138–140). Bisphosphonate prodrugs already depicted some effect in combination with adoptive cell transfer in an animal model of bladder carcinoma and human fibrosarcoma (138, 139).

Nano-technology based carriers for N-BP delivery (141) as well as lipophilic bisphosphonate (60, 142, 143) and synthetic nucleotide pyrophosphates (110) are additional pharmacotherapeutic strategies that may improve Vγ9Vδ2 T-cell immunotherapy in the future.

### Butyrophilin 3A (BTN3A)

More recently, Butyrophilin 3A (BTN3A, CD277) was described as essential for γδ T-cell activation by direct PAgs (144, 145). BTN3A belongs to the important B7 family of co-stimulatory molecules (146) and consists of three isoforms: BTN3A1, BTN3A2, and BTN3A3. BTN3A2 differs as it lacks an intracellular B30.2 domain that is needed for PAg recognition. However, when using the mouse anti-human-CD277 antibody clone 20.1 directed against an extracellular domain, all BTN3A isoforms support Vγ9Vδ2 T-cell activation (144). The molecular details of signal transduction are a current research topic and matter of debate, especially regarding two different models: originally, the “antigen presenting model” by Vavassori et al. (145) assuming that CD277 and the TCR interact directly following PAg binding to an extracellular CD277 domain. Recent experimental evidence rather supports a second, so called “allosteric model” by Harly et al., postulating that PAgs interact with the intracellular B30.2 domain of CD277 (147) either directly (148) or indirectly (149, 150) and induce a conformational change that is transferred to the extracellular parts of the CD277 molecule (147, 151). PAg sensing may additionally involve molecules like Rho-GTPase (152) or Periplakin and is modulated by mechanisms enabling transmembranous PAg transport or *via* hydrolyzation of PAgs by ecto-ATPase CD39 (106, 153).

Development of mouse anti-human-CD277 antibodies has been very useful in deciphering the activation processes of Vγ9Vδ2 T-cells (144, 154) and also holds therapeutic potential. The mode of action of these antibodies was proven to be downstream and independent of IPP (144, 149). Furthermore, activating anti-CD277 clone 20.1 has similar but not identical stimulatory capabilities compared with PAg stimulation (155) and might be restricted to certain Vγ9Vδ2 T-cells with specific complementarity-determining region sequences of the TCR (156). Still, anti-CD277 antibodies might outperform N-BPs or other metabolic sensitizers in target cells that fail to internalize drugs or which have decreased mevalonate pathway activity. It was shown that anti-CD277 antibodies enhance anti-tumor functions of Vγ9Vδ2 T-cells *in vitro* (144) and in a xenotransplant mouse model of human acute myeloid leukemia (AML) (157). We also observed that primary chronic lymphatic leukemia (CLL) cells are hardly affected by ZOL sensitization become lysed by Vγ9Vδ2 T-cells following their incubation with activating anti-CD277 antibody (158). Unfortunately, antibodies with a murine background seem inappropriate for clinical use and development of a humanized version or a human homolog of the clone 20.1 antibody has not been reported. A further drawback is the widespread expression of the CD277 molecule in human tissues (146, 159), which is why additional strategies for enhancement of selectivity might be required. One solution could be the development of antibody constructs combining both CD277 activating and tumor-antigen specificity.

### Other Agents

Therapeutic specificity might also be achieved by targeting tumor cell specific metabolic alterations. Therefore, we tested whether the pyruvate dehydrogenase activator dichloroacetate (DCA) might improve Vγ9Vδ2 T-cell anti-tumor functions *in vitro*. DCA inhibits aerobic glycolysis, malignant cell proliferation and indirectly facilitates mitochondrial oxidative decarboxylation of pyruvate to acetyl-coenzyme A (160). Indeed, we found that DCA + ZOL treated leukemia cell lines induce higher IFN-γ production by Vγ9Vδ2 T-cells compared with ZOL treatment alone. We also suspected that DCA increases the supply of metabolites upstream of IPP and therefore increases PAg accumulation when combined with ZOL (161). Still, alternative explanations are possible as DCA decreases tumor cells’ LA production (160) and LA can directly inhibit several immune functions. Tumor LA efflux is, therefore, an attractive target and could be targeted by inhibition of lactate transporters and nonsteroidal anti-inflammatory drugs (NSAID) (101). Concerning potential anti-tumor effects of NSAIDs, the use of indomethacin as well as specific cyclooxygenase-2 inhibitors resulted in an increase of Vγ9Vδ2 T-cell dependent tumor cell lysis. If this observation is connected to LA release has not been investigated but was attributed to the inhibition of prostaglandin effects (162). Finally, the enzymes CD39 and CD73 that regulate ATP/adenosine balance and thereby the function of immune cells might represent interesting targets for immunotherapy (163). Here, CD39 might be of special interest in the context of Vγ9Vδ2 T-cell therapy as it was shown to be capable of PAg hydrolyzation (164).

### Summary

Adoptive transfer of *ex vivo* cultured cells and various combinations of N-BPs, BrHPP, and IL-2 have demonstrated clinical effects but are rather disappointing compared to the promising pre-clinical results. The discrepancy suggests that the *in vivo* characteristics of stimulated Vγ9Vδ2 T-cells are still insufficiently understood. To overcome the current limitations, we need to learn more about differentiation and functionality of PAg activated γδ T-cells, its subpopulations and migration patterns. PAgs and N-BPs with improved pharmacokinetics and potency are very promising new developments, but their toxicity profile and clinical effectiveness have yet to be established. A breakthrough would be the development of PAg or N-BP analogs with strong molecular tumor cell specificity.

Beside these innovations, we should search for additional tumor-specific transport mechanisms and metabolic peculiarities. A good example for the exploitation of a “metabolic weak spot” in cancer is the use of asparaginase in acute lymphatic leukemia (165). We need to identify such targets in the context of γδ T-cell sensing and will hopefully be able to design specific and effective compounds at least for certain types of cancer. Finally, we should consider the metabolic needs of immune cells as well. They may also rely on mevalonate pathway products or upregulate aerobic glycolysis following activation (166) and therefore become negatively affected by certain therapeutic interventions.

## Targeting Activating and Inhibitory Receptors

### NKG2D and Its Ligands

In innate immune responses mediated by NK-cells, NKG2D serves as a primary activating receptor and ligand binding triggers cytotoxicity and cytokine production (167–169). In humans, one NKG2D homodimer assembles with four DAP10 adaptor proteins that become phosphorylated upon ligand binding and activation (170). Ligands from distinctive families, the MHC class I polypeptide related sequence A (MICA) and B (MICB) and the cytomegalovirus UL16-binding protein (ULBP) family bind NKG2D even though they share little sequence similarity (171). The expression of NKG2D ligands (NKG2DL) is induced or upregulated primarily in tissues of epithelial origin, as a result of cellular stresses such as viral infection, malignant transformation, or classical heat shock (172, 173). All NKG2DLs are not functionally equivalent and can enable immune cells to recognize of a broad range of different types of infections and indicate malignant transformation in different tissues (170, 171, 174).

NKG2D is also expressed by γδ T-cells and provides important (co-)stimulatory signals in T-cell-mediated immune responses by amplifying T-cell cytokine production, proliferation, and cytotoxicity *in vitro* (52, 98, 169, 175). The NKG2D pathway is also relevant in the context of N-BP treatment and the expression of ULBP1 was found correlated with the sensitivity of AML blasts toward TCR-mediated killing by Vγ9Vδ2 T-cells (114). Additionally, the results of Wrobel et al. indicated that the NKG2D pathway is involved in anti-tumor effects of γδ T-cells against melanoma and various epithelial cancers (55).

### MICA-Polymorphism and Soluble MIC (sMIC)

The general concept is that cell stress and transformation increase the expression of MICA antigens and activate immune cells *via* NKG2D. However, MICA is a highly polymorphic human stress antigen and Shafi et al. showed that MICA coding sequence polymorphisms substantially affected RNA and protein expression (176). Some examined individuals showed better response to higher, others to lower MICA expression, and challenging the concept of an invariable direct correlation between stress molecule abundance and immune cell activation (176, 177).

Tumors also adopt evasion strategies, like shedding of free or the exosome form of MICA/MICB. These released molecules can inhibit immune effector cells due to interaction with NKG2D (178). Märten et al. found elevated levels of sMIC levels in sera of patients with pancreatic carcinoma correlated with tumor stage. The cytotoxic response of immune toward tumor cells was found impaired with in the presence of high sMIC levels but restored by neutralization of sMIC (179).

#### Temozolomide (TMZ) and Other Chemotherapeutics

Glioblastoma multiforme (GBM) is an extremely aggressive brain tumor, which is not very sensitive to either classical chemotherapy or immunotherapeutic approaches. Lamb et al. showed that *ex vivo* expanded γδ T-cells recognize malignant glioma *via* NKG2DL and lyse glioma cell lines and primary GBM specimens. Additionally TMZ, a DNA methylating chemotherapeutic agent licensed for GBM therapy, increased NKG2DL also on TMZ-resistant glioma cells. They also demonstrated that immune effector cells can be genetically modified to resist the toxicity of TMZ without changing their phenotype or their cytotoxicity against GBM target cells (180). Similarly, Chitadze et al. investigated the NKG2DL system in different GBM cell lines and confirmed that TMZ increased the cell surface expression of NKG2DL and sensitizes GBM cells to γδ T-cell mediated lysis. TMZ might therefore enhance the potential of adoptive transfer of *ex vivo* expanded γδ T-cells for glioblastoma treatment (181, 182).

Dacarbazine is a cytotoxic drug used for treatment of Hodgkin’s lymphoma and melanoma. Although dacarbazine does not directly affect immune cells, it triggers the upregulation of NKG2DL on tumor cells, leading to NK-cell activation and IFN-γ secretion in mice and humans (183). Apart from TMZ and dacarbazine, studies suggest that other chemotherapeutics, like fluorouracil, doxorubicin, or vincristine sensitize tumor cell lines toward a NKG2D-dependent cytotoxic activity of Vγ9Vδ2 T-cell (184, 185). This could be a target cell or drug specific phenomenon as we were unable to boost γδ T-cell induced lysis of several leukemia cell lines with other cytostatic drugs (186).

#### Bortezomib and Epigenetic Drugs

Niu et al. reported that multiple myeloma (MM) cells can be sensitized toward killing by γδ T-cells and NK-cells using low-dose bortezomib. Additionally, bortezomib increases the expression of NKG2D and induces apoptosis of MM-cells, but not γδ T-cells and NK-cells (187). Treatment with 5-azacytidine, its derivate decitabine or histone deacetylase inhibitors may also increase the expression of NKG2DL in different types of malignancies prompting Bhat et al. to consider those epigenetic drugs a promising approach in γδ T-cell immunotherapy (188). Suzuki et al. evaluated possible additive effects of valproic acid (VPA), a histone deacetylase inhibitor, on γδ T-cell mediated cytotoxicity against bladder cancer cell lines TCCSUP and 253J (189). VPA did increase expression of NKG2DL and sensitivity toward cytolysis by γδ T-cells for both cancer cell types, whereas ZOL pre-treatment was only effective against TCCSUP. 253J cells were preferentially engaged *via* NKG2D-NKG2DL interaction, while TCCSUP cells were mainly recognized through the γδ TCR (189). Chávez-Blanco et al. showed that hydralazine in combination with VPA increase the expression of MICA and MICB ligands by target cells, as well as NK-cell cytotoxicity *via* NKG2D. Additionally it reduces the shedding of MIC molecules to the supernatant (190). Satwani et al. incubated acute lymphoblastic leukemia and non-Hodgkin lymphoma cell lines for 24 h with 10 ng/mL of romidepsin (191). They demonstrated an approximately 50- to 1,300-fold increase in the number of cells positive for the surface expression of MICA/B in these cell lines. They further demonstrated a significant increase in NK-cell-mediated *in vitro* cytotoxicity (191).

### Inhibitory Receptors

The development of immune checkpoint inhibitors targeting the cytotoxic T-lymphocyte-associated Protein 4 (CTLA4) or programmed cell death protein 1 (PD-1) and its ligand (PD-L1) has substantially extended the possibilities of immunotherapy. These substances are able to induce enduring remissions in a considerable subset of patients with treatment refractory types of cancer, for example melanoma, non-small cell lung cancer, and Hodgkin’s lymphoma (192). Considering their clinical significance, relatively little is known about the role of γδ T-cells in immune checkpoint therapy and also regarding the role of inhibitory axes for γδ T-cell biology.

#### Programmed Cell Death Protein 1

Programmed cell death protein 1 is a key inhibitory receptor in inflammation, responsible for induction of tolerance, and immunosuppression in cancer (193). Following interaction with its ligands programmed death-ligand 1/2, the PD-1 receptor inhibits TCR and PI3K/AKT signaling and decreases proliferation and IL-2 release (194). It is interesting that both the PD-Ls and the CTLA4 ligands (CD80 and CD86) are members of the B7 family of proteins and therefore interrelated to BTN3A/CD277. Several types of malignancies have a relevant susceptibility to therapeutic PD-1/PD-L1 blockade, but it is barely predictable which individual patient will respond. The initially assumed direct relationship between tumor cell expression of PD-Ls and response rate following therapeutic PD-1 blockade might not be universally valid and the strength of PD-1 dependent immunosuppression is influenced by the topographic organization of the tumor microenvironment (195).

An early *in vitro* study addressed the expression profile and functionality of PD-1/PD-L1 in γδ T-cells following stimulation with HMBPP and suggested that the PD-1/PD-L1 axis is important for regulation of anti-tumor mechanisms of γδ T-cells (196). Later it was found that PD-1 expression is more frequent on Vδ1, compared with Vδ2 T-cells (197) and equably distributed over several functionally distinctive subsets of Vγ9Vδ2 T-cells (44). A report that *ex vivo* cultivated Vδ2 T-cells depict stable, low cell surface expression of PD-1 following adoptive transfer (198) might fit the observations that PD-1 is only temporarily upregulated following *in vitro* stimulation as it has been reported both for HMBPP and ZOL (196, 198). Vδ2 T-cells derived from neonates may behave differently as they depict prolonged PD-1 expression following activation and function as a regulator of tumor necrosis factor-α (TNF-α) production and cell degranulation, both being part of fetal inflammatory response (199).

Programmed cell death protein 1 expression might contribute to insufficient expansion of Vγ9Vδ2 T-cells in cancer patients, as a diminished response to PAg stimulation was demonstrated in bone marrow derived Vγ9Vδ2 T-cells from patients with MM. Such cells depicted a significantly increased PD-1 expression and were located in proximity to PD-L1+ MM-cells and myeloid-derived suppressor cells (200). Additional treatment with PD-1 antibody resulted in a twofold increase in proliferative response and an increased mobilization of CD107a following ZOL stimulation *in vitro* (200). Beside the bone marrow of MM patients, PD-1 positive γδ T-cells were also found in neuroblastoma infiltrated bone marrow (201).

#### Other Inhibitory Receptors

Alongside PD-1 several other inhibitory molecules are currently investigated regarding their function in limiting anti-tumor responses and potential therapeutic prospects (202). This is of special interest as there are indications for compensatory upregulation of alternative inhibitory receptors during anti-PD-1 therapy (203). Examples are the B- and T-lymphocyte attenuator (BTLA), CTLA4, T-cell immunoglobulin and mucin-domain containing-3 (TIM-3), and lymphocyte activation gene-3 (LAG-3) and their respective ligands.

B- and T-lymphocyte attenuator was suggested to inhibit late phases of immune reactions and has structural and functional similarities to PD-1 and CTLA4 (204). It is expressed on Vγ9Vδ2 T-cells and engagement by its ligand, the herpesvirus entry mediator, reduced activation, proliferation, and anti-lymphoma response (205). Differing from PD-1 expression kinetics (196, 198), BTLA is initially downregulated following stimulation with PAgs but upregulated upon IL-7 treatment (205).

Compared with PD-1 and BTLA, even less is known concerning the functional implications of CTLA4, LAG-3, and TIM-3 on γδ T-cells. Melanoma patients with a higher ratio of Vδ1 to total γδ T-cells had poorer overall survival and *vice versa* higher frequencies of Vδ2 cells were associated with longer survival in a study using CTLA4 inhibitory antibody ipilimumab (16). Expression of LAG-3 indicates inhibition of PD-1 + T-cells in the tumor tissue and poorer prognosis in follicular lymphoma (206). From studies examining distinctive T-cell populations, we know that CTLA4 can inhibit T-cell activity *via* signaling mechanisms distinctive from PD-1 (207), but we still lack mechanistic studies conclusively demonstrating CTLA4 expression and function for Vγ9Vδ2 T-cells. In women with pre-eclampsia γδ T-cells with low TIM-3 expression depict a higher IFN-γ production (208) and in the context of malaria infection a high TIM-3 level was found correlated with reduced pro-inflammatory cytokine production (209). Similar to anti-PD-L1 antibodies, the inhibition of the TIM-3 ligand galactine-9 that is expressed by γδ T-cells, increases tissue infiltration by αβ T-cells in a pancreatic tumor model (5).

### Summary

The referred data provide interesting prospects to enhance immunotherapy by means of modulating the expression of NKG2DL. Even though several of the referred effects were shown for NK-cells, these strategies might also apply for sensitizing tumor cells toward γδ T-cell dependent cytotoxicity. Negative aspects like possible adverse effects on immune cell functionality or tumor escape mechanisms like sMIC and MICA-polymorphism need to be considered in future studies.

The physiological relevance of the currently known inhibitory receptors for γδ T-cells biology remains vague and additional observational and experimental studies are required. Based on the current evidence we assume that PD-1 is important for regulation of Vγ9Vδ2 T-cell functionality under specific conditions only, for example in an immunosuppressive tumor microenvironment. In inflammation and in the tumor microenvironment, γδ T-cells can become inhibited *via* PD-1 and also inhibit other PD-1 + immune cells *via* PD-L1 expression (5, 210). However, inhibitory effects of PD-1 may be overruled upon strong (co-)stimulation, for example *via* the TCR or with IL-2. Beside the local tissue distribution of receptors and ligands, expression kinetics are important to understand the function of the inhibitory receptors for immune homeostasis. Unfortunately, many studies do not distinguish whether tissue infiltrating T-cells are αβ or γδ T-cells in the first place. Combination therapy of adoptive transfer or *in vivo* stimulation of γδ T-cells with PD-1, PD-L1, CTLA4, or BTLA antibodies therefore seems feasible but the pre-clinical rational is currently not well established.

## ADCC and Antibody Constructs

Cytotoxicity of γδ T-cells against target cells can be significantly enhanced using specific monoclonal antibodies (mAbs) that induce ADCC. ADCC of γδ T-cells is thought to depend on Fc-γ receptor III (CD16) as it has been demonstrated that anti-CD19 antibody triggered CD107a, IFN-γ, and TNF-α expression is correlated to the amount of CD16+ γδ T-cells in an *in vitro* cytotoxicity assay (211). Furthermore, γδ T-cell mediated ADCC increases with higher numbers of CD16+ γδ T-cells (212) and was found inhibited with CD16 blocking antibodies (213). CD16 expression is usually low in unstimulated γδ T-cells, but increases following activation, for example with PAgs (213, 214).

### B-Cell Malignancies

#### Rituximab

Several lymphoma and B-cell lineage leukemia subtypes were studied using stimulated γδ T-cells in combination with monoclonal anti-CD20 antibodies (212–216). Tokuyama et al. found RTX to increase the killing of several lymphoma cell lines and to improve ADCC of γδ T-cells against CLL and autologous follicular lymphoma cells (213). Furthermore, BrHPP stimulated γδ T-cells demonstrated stronger CD107a expression and increased ADCC toward individual B-cell lymphoma cell lines and patient CLL cells in combination with anti-CD20 antibodies (214). One single clinical phase I/IIa study used RTX plus BrHPP and IL-2 for *in vivo* stimulation of γδ T-cells in patients with relapsed follicular lymphoma (89). Altogether, 45 patients were treated according to protocol and the treatment was generally well tolerated, with low grade pyrexia being the most common side effect (89). Despite the 45% overall response rate (26% complete response) (89), it seems like development of BrHPP containing therapies is no longer pursued by the company in charge.

#### Second Generation Anti-CD20 Antibodies and Anti-CD52

The newer anti-CD20 antibodies ofatumumab and obinutuzumab were also tested regarding the efficacy inducing ADCC in connection with γδ T-cells (215). Obinutuzumab is an Fc engineered type II monoclonal antibody (217) and causes an increased secretion of perforin and IFN-γ compared to RTX and ofatumumab. Accordingly, the highest ADCC against B-cell lymphoma cell lines and primary follicular lymphoma cells was found for obinutuzumab (215). Similar to anti-CD20 antibodies, Gertner-Dardenne found alemtuzumab, an anti-CD52 antibody, to increase γδ T-cell dependent ADCC against lymphoma cell lines (214).

### Solid Tumors

#### Breast Cancer

Two groups investigated whether the human epidermal growth factor receptor 2 (HER2/neu) specific antibody trastuzumab enhances γδ T-cell dependent ADCC toward breast cancer cell lines *in vitro* (63, 213). The addition of trastuzumab greatly increased lysis of HER2/neu overexpressing cell lines, whereas there was no change in a HER2/neu negative cell line (213). The extent of ADCC was increased with higher density of HER2/neu expression. Anti-tumor activity was confirmed in an animal model with SCID Beige mice. Here, the tumor growth was more efficiently inhibited by a combination treatment with γδ T-cells and trastuzumab compared to treatment with trastuzumab or γδ T-cells alone (63).

#### Neuroblastoma and Ewing’s Sarcoma

Both in neuroblastoma and in Ewing’s sarcoma, the disialoganglioside specific antibody ch14.18/CHO increased γδ T-cell mediated ADCC *in vitro* (124, 218). This finding was confirmed in an advanced immunodeficient mouse model, where *ex vivo* stimulated and adoptively transferred γδ T-cells with simultaneous administration of ch14.18/CHO antibody impaired tumor growth more efficiently than single antibody or sole γδ T-cells treatment (124).

### Antibody Constructs and Nanobodies

Antibody constructs have been studied in both lymphoma and solid tumor models. Seidel et al. used the Fc modified CD19 antibody 4G7SDIE as a backbone for bispecific CD19-CD16 and CD19-CD3 antibody constructs (211). Although no direct comparison between unaltered antibodies and the antibody constructs was made, the constructs proofed active in inducing cytotoxic reactions by γδ T-cells. Schiller et al. went one step further and engineered a so called “single chain triplebody,” called SPM-1, that consists of three single chain antibody fragments (CD19-CD19-CD16) (219). Indeed, SPM-1 induced a higher lysis compared to 4G7SDIE. A comparable approach is a recombinant construct consisting of a CD20 single-chain fragment variable (scFV) linked to MICA or ULBP2 which enhances cytotoxicity of stimulated γδ T-cells against CD20+ lymphoma cell lines and primary CLL cells *via* NKG2D (220). Oberg et al. designed two bispecific antibodies that bind either CD3 or the Vγ9 TCR-chain on γδ T-cells and Her2/neu expressed by pancreatic adenocarcinoma cells (221). Both antibodies enhanced γδ T-cell mediated cytotoxicity and adoptive transfer of γδ T-cells combined with [(HER2)2xVγ9] antibody therapy inhibited growth of pancreatic cancer in a SCID Beige mouse model (221). Furthermore, Hoh et al. demonstrated improved anti-tumor effects against hepatocellular carcinoma and hepatoblastoma cells with MT110, an epithelial cell adhesion molecule EpCAM/CD3 bispecific T-cell engager antibody, compared to the anti-EpCAM antibody adecatumumab (222). Zhang et al. utilized a bifunctional fusion protein (anti-CD3 single-scFV/-NKG2D) that binds NKG2DL+ tumor cells and recruits and stimulates T-cells *via* CD3 (223). This fusion protein was able to stimulate IFN-γ production by T-cells, increased cytotoxic reaction against NKG2DL+ tumor cells *in vitro* and promoted survival in a murine lymphoma model (223).

Another innovative approach is the use of so called nanobodies, a single heavy chain fragment. They bind highly selective to the Vγ9Vδ2 chain and elicited either inhibiting or activating reactions from γδ T-cells (224, 225). Although no data on cytotoxic features against tumor cells are available, it seems to be a promising approach to a selective modulation of Vγ9Vδ2 T-cell activity.

### Summary

Monoclonal antibodies combine high target specificity with a favorable toxicity profile, but often depict limited activity when used as single agents. Therefore, combination with γδ T-cells is a promising concept for cancer immunotherapy. There are many mAbs for various hematological and non-hematological malignancies in clinical use already and more are currently in pre-clinical or early clinical development. Several such mAbs are promising combination partners as they show a uniformly strong enhancement in γδ T-cell mediated cytotoxicity. However, results of the only clinical study in this regard, which used RTX plus *in vivo* stimulation of γδ T-cells fell short of expectations. With the advent of new and Fc optimized antibodies and more specifically stimulated γδ T-cells, a higher effectivity might be achievable.

## Counteracting Pro-Tumor Effects

The local interplay of malignant, immune and stroma cells *via* direct cellular interactions and soluble factors characterizes the tumor microenvironment. Under these conditions, infiltrating immune cells can be suppressed and therapeutic activation may even unfold unintended tumor-promoting effects. Beside macrophages and regulatory T-cells (70, 96), IL-17-producing γδ T-cells (γδ T17 cells) are often suggested as important local mediators of tumor progression as repetitively demonstrated in animal models (226–228). It is possible to induce IL-17 production in human cells γδ T-cells *in vitro* (229) and γδ T17 cells were described in the human tumor microenvironments (7, 230) where they have been found inversely correlated with survival and associated with increased stage in breast (6) and colorectal cancer (7). It is important to note that not all studies differentiated between Vδ2 and non-Vδ2 cells or other γδ T-cell subclasses but it seems likely that both, Vδ2 but mainly the non-Vδ2 cells produce IL-17 (7). Direct proof is lacking, but it has been suggested that γδ T-cells can be changed toward an IL-17 producing phenotype by means of the tumor microenvironment (229, 231). Beside IL-17, vascular endothelial growth factor (VEGF) and granulocyte-macrophage colony-stimulating factor are predominately recognized as pro-tumor factors in the microenvironment, but it may not be reasonable to attribute an exclusive pro- or anti-tumor effect to any signal protein, cytokine, cell type or receptor-ligand interaction. For example VEGF facilitates neo-angiogenesis and immunosuppressive effects (232, 233) but also promotes tissue trafficking of different leukocytes (234, 235). The use of immunostimulatory drugs can induce unexpected changes in VEGF levels, as we observed an increase in VEGF serum levels following treatment with ZOL plus low-dose IL-2 in cancer patients (72). Pro-angiogenic factors like VEGF play an important pro-tumor role and predict poor clinical response to certain types of immunotherapy (72, 236). We recently described that following stimulation with IL-2 local lymphocyte-monocyte interactions regulate VEGF homeostasis *via* release of VEGF and soluble VEGF receptor 1 in a time-dependent manner *in vitro* (237). Potential pro-tumor factors and cells could be additionally targeted in combination with γδ T-cell therapy, for example *via* VEGF or IL-17 antagonists. VEGF antibodies are already widely used as cancer therapeutics making clinical studies investigating such a combination therapy feasible. The modest clinical effects of anti-angiogenic strategies call for a more fundamental analysis of VEGF signaling in the tumor microenvironment and the contribution of immune cells to these processes. The same also applies for other factors like IL-17.

Finally, both pro- and anti-tumor effects are mediated locally, as a consequence the *in vivo* efficacy of Vγ9Vδ2 T-cells will depend on their ability to infiltrate into the relevant tissues. Unfortunately we have little information concerning the capacity of activated γδ T-cells to reach the tumor in humans. One single clinical study demonstrated that autologous, *ex vivo* stimulated γδ T-cells predominately migrate to lung, liver and spleen and could also be detected in individual tumor sites (84). Whether or not an effector cell is capable of tissue homing might be predicted by expression of chemokine receptors, selectins and other cell adhesion molecules. Expression of these molecules however depends on γδ T-cells subpopulation and differentiation status (43, 238, 239).

## Conclusion

**Figure 1:**
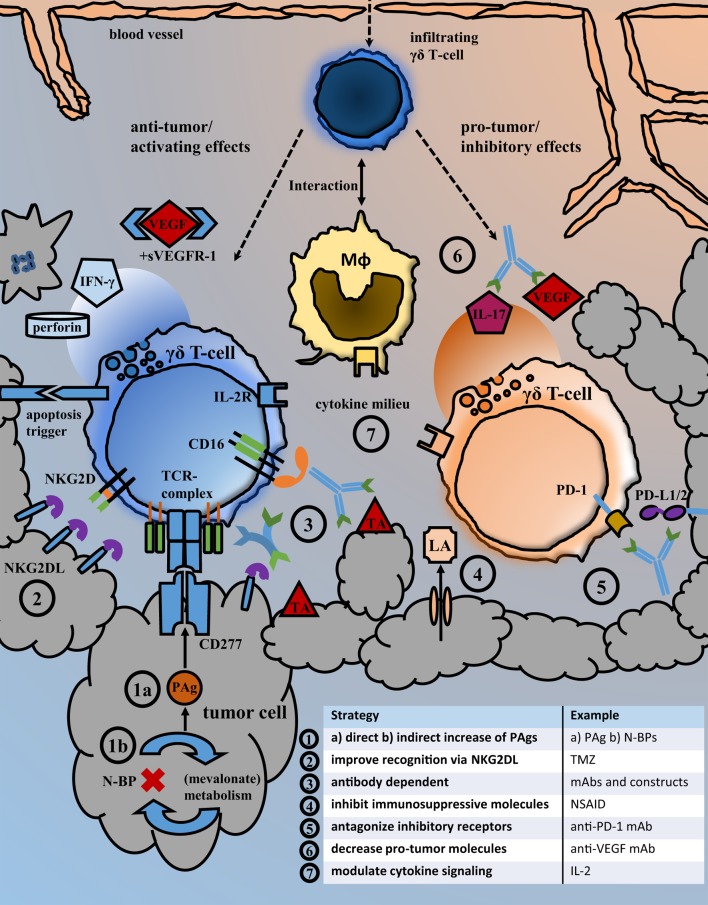
Strategies for the inhibition of pro-tumor and the enhancement of anti-tumor effects. Overview of the local tumor microenvironment that illustrates important immune cell interactions and exemplary types of therapeutic interventions facilitating anti-tumor activity. Following their migration from blood to tissue, γδ T-cells may interact with macrophages and exhibit local pro- but also anti-tumor effects. Possible therapeutic strategies aiming to improve the recognition and killing of cancer cells by γδ T-cells as well as those intended to antagonize immunosuppressive receptor signaling and molecules are listed under points 1–7. Abbreviations: BrHPP, bromohydrin pyrophosphate; DCA, dichloroacetate; (G)M-CSFR, (granulocyte-)macrophage colony-stimulating factor receptor; IFN-γ, interferon-γ; IL-2R, interleukin-2 receptor; LA, lactic acid; mAb, monoclonal antibody; Mφ, macrophage/monocyte lineage cell; N-BP, amino-bisphosphonates; NKG2DL, NKG2D ligands; NSAID, nonsteroidal anti-inflammatory drugs; PAg, phosphoantigens; PD-1, programmed cell death protein 1; PD-L1/2, programmed death-ligand 1/2; sVEGFR-1, soluble vascular endothelial growth factor receptor; TA, tumor antigen; TCR-complex, T-cell receptor complex; TMZ, temozolomide; VEGF, vascular endothelial growth factor.

The results from pre-clinical research and individual clinical responses to γδ T-cell therapy encourage to carry on studying γδ-T-cell biology and aim to improve γδ T-cell related anti-cancer therapies. The question is, how the manifold observations on cellular mechanisms can help to establish better anti-cancer strategies and which drugs have an actual translational perspective. An overview on current γδ T-cell dependent therapeutic strategies and immune cell interactions in the tumor microenvironment is given in Figure 1. The use of mAb in combination with activated γδ T-cells is strikingly effective *in vitro*. Still the results from *in vivo* experiments did not always keep up with such expectations and the results of the only clinical trial did not proof superior to mAb monotherapy. We will need a thorough understanding of Vγ9Vδ2 T-cell subpopulations and their functional differences and must learn how to influence differentiation and prevent exhaustion. Our knowledge regarding the migration and tissue infiltration of Vγ9Vδ2 T-cells *in vivo* is still sparse, as is the understanding of pro- and anti-tumor mechanisms and cellular interactions in the tumor microenvironment. The establishment of better models could help deciphering those local and time-dependent processes. While the relevance of metabolic changes for immune and cancer cell function is now increasingly acknowledged, we need to learn how immune cells detect and respond to such changes. Reactivity to PAg by Vγ9Vδ2 T-cell may serve as an example, but we should be able to target even more specific tumor characteristics with cellular or combination therapy in the future.

## Author Contributions

TH wrote the manuscript and prepared the figure. MS and DP drafted sections and edited the manuscript. MW structured and edited the manuscript. All authors read and approved the submitted version.

## Conflict of Interest Statement

The authors declare that the research was conducted in the absence of any commercial or financial relationships that could be construed as a potential conflict of interest.

## References

[1] Girardi M Oppenheim DE Steele CR Lewis JM Glusac E Filler R Regulation of cutaneous malignancy by gammadelta T cells. Science (2001) 294(5542):605–9. 10.1126/science.1063916 11567106

[2] Silva-Santos B Serre K Norell H [Gamma][delta] T cells in cancer. Nat Rev Immunol (2015) 15(11):683–91. 10.1038/nri3904 26449179

[3] Bialasiewicz AA Ma JX Richard G. Alpha/beta- and gamma/delta TCR(+) lymphocyte infiltration in necrotising choroidal melanomas. Br J Ophthalmol (1999) 83(9):1069–73. 10.1136/bjo.83.9.1069 10460778 PMC1723174

[4] Cordova A Toia F La Mendola C Orlando V Meraviglia S Rinaldi G Characterization of human gammadelta T lymphocytes infiltrating primary malignant melanomas. PLoS One (2012) 7(11):e49878 10.1371/journal.pone.0049878 23189169 PMC3506540

[5] Daley D Zambirinis CP Seifert L Akkad N Mohan N Werba G Gammadelta T cells support pancreatic oncogenesis by restraining alphabeta T cell activation. Cell (2016) 166(6):1485–99.e15. 10.1016/j.cell.2016.07.046 27569912 PMC5017923

[6] Ma C Zhang Q Ye J Wang F Zhang Y Wevers E Tumor-infiltrating gammadelta T lymphocytes predict clinical outcome in human breast cancer. J Immunol (2012) 189(10):5029–36. 10.4049/jimmunol.1201892 23034170 PMC4832413

[7] Wu P Wu D Ni C Ye J Chen W Hu G GammadeltaT17 cells promote the accumulation and expansion of myeloid-derived suppressor cells in human colorectal cancer. Immunity (2014) 40(5):785–800. 10.1016/j.immuni.2014.03.013 24816404 PMC4716654

[8] Zocchi MR Ferrarini M Migone N Casorati G. T-cell receptor V delta gene usage by tumour reactive gamma delta T lymphocytes infiltrating human lung cancer. Immunology (1994) 81(2):234–9. 8157272 PMC1422308

[9] Bas M Bier H Schirlau K Friebe-Hoffmann U Scheckenbach K Balz V Gamma-delta T-cells in patients with squamous cell carcinoma of the head and neck. Oral Oncol (2006) 42(7):691–7. 10.1016/j.oraloncology.2005.11.008 16527515

[10] Groh V Rhinehart R Secrist H Bauer S Grabstein KH Spies T. Broad tumor-associated expression and recognition by tumor-derived gamma delta T cells of MICA and MICB. Proc Natl Acad Sci U S A (1999) 96(12):6879–84. 10.1073/pnas.96.12.6879 10359807 PMC22010

[11] Oberg HH Peipp M Kellner C Sebens S Krause S Petrick D Novel bispecific antibodies increase gammadelta T-cell cytotoxicity against pancreatic cancer cells. Cancer Res (2014) 74(5):1349–60. 10.1158/0008-5472.CAN-13-0675 24448235

[12] Tosolini M Pont F Poupot M Vergez F Nicolau-Travers ML Vermijlen D Assessment of tumor-infiltrating TCRVgamma9Vdelta2 gammadelta lymphocyte abundance by deconvolution of human cancers microarrays. Oncoimmunology (2017) 6(3):e1284723 10.1080/2162402x.2017.1284723 28405516 PMC5384348

[13] Gentles AJ Newman AM Liu CL Bratman SV Feng W Kim D The prognostic landscape of genes and infiltrating immune cells across human cancers. Nat Med (2015) 21(8):938–45. 10.1038/nm.3909 26193342 PMC4852857

[14] Meraviglia S Lo Presti E Tosolini M La Mendola C Orlando V Todaro M Distinctive features of tumor-infiltrating gammadelta T lymphocytes in human colorectal cancer. Oncoimmunology (2017) 6(10):e1347742 10.1080/2162402X.2017.1347742 29123962 PMC5665062

[15] Zocchi MR Ferrarini M Rugarli C. Selective lysis of the autologous tumor by delta TCS1+ gamma/delta+ tumor-infiltrating lymphocytes from human lung carcinomas. Eur J Immunol (1990) 20(12):2685–9. 10.1002/eji.1830201224 1702723

[16] Wistuba-Hamprecht K Martens A Haehnel K Geukes Foppen M Yuan J Postow MA Proportions of blood-borne Vdelta1+ and Vdelta2+ T-cells are associated with overall survival of melanoma patients treated with ipilimumab. Eur J Cancer (2016) 64:116–26. 10.1016/j.ejca.2016.06.001 27400322 PMC5201188

[17] Pressey JG Adams J Harkins L Kelly D You Z Lamb LS Jr In vivo expansion and activation of gammadelta T cells as immunotherapy for refractory neuroblastoma: a phase 1 study. Medicine (Baltimore) (2016) 95(39):e4909 10.1097/MD.0000000000004909 27684826 PMC5265919

[18] Wilhelm M Kunzmann V Eckstein S Reimer P Weissinger F Ruediger T Gammadelta T cells for immune therapy of patients with lymphoid malignancies. Blood (2003) 102(1):200–6. 10.1182/blood-2002-12-3665 12623838

[19] Yi Y He HW Wang JX Cai XY Li YW Zhou J The functional impairment of HCC-infiltrating gammadelta T cells, partially mediated by regulatory T cells in a TGFbeta- and IL-10-dependent manner. J Hepatol (2013) 58(5):977–83. 10.1016/j.jhep.2012.12.015 23262246

[20] Gaafar A Aljurf MD Al-Sulaiman A Iqniebi A Manogaran PS Mohamed GE Defective gammadelta T-cell function and granzyme B gene polymorphism in a cohort of newly diagnosed breast cancer patients. Exp Hematol (2009) 37(7):838–48. 10.1016/j.exphem.2009.04.003 19446661

[21] Zheng BJ Ng SP Chua DT Sham JS Kwong DL Lam CK Peripheral gamma delta T-cell deficit in nasopharyngeal carcinoma. Int J Cancer (2002) 99(2):213–7. 10.1002/ijc.10326 11979436

[22] Sicard H Ingoure S Luciani B Serraz C Fournie JJ Bonneville M In vivo immunomanipulation of V gamma 9V delta 2 T cells with a synthetic phosphoantigen in a preclinical nonhuman primate model. J Immunol (2005) 175(8):5471–80. 10.4049/jimmunol.175.8.5471 16210655

[23] Dieli F Vermijlen D Fulfaro F Caccamo N Meraviglia S Cicero G Targeting human {gamma}{delta} T cells with zoledronate and interleukin-2 for immunotherapy of hormone-refractory prostate cancer. Cancer Res (2007) 67(15):7450–7. 10.1158/0008-5472.CAN-07-0199 17671215 PMC3915341

[24] Godder KT Henslee-Downey PJ Mehta J Park BS Chiang KY Abhyankar S Long term disease-free survival in acute leukemia patients recovering with increased gammadelta T cells after partially mismatched related donor bone marrow transplantation. Bone Marrow Transplant (2007) 39(12):751–7. 10.1038/sj.bmt.1705650 17450185

[25] Hayday AC Saito H Gillies SD Kranz DM Tanigawa G Eisen HN Structure, organization, and somatic rearrangement of T cell gamma genes. Cell (1985) 40(2):259–69. 10.1016/0092-8674(85)90140-0 3917858

[26] Loh EY Lanier LL Turck CW Littman DR Davis MM Chien YH Identification and sequence of a fourth human T cell antigen receptor chain. Nature (1987) 330(6148):569–72. 10.1038/330569a0 2825032

[27] Munoz-Ruiz M Perez-Flores V Garcillan B Guardo AC Mazariegos MS Takada H Human CD3gamma, but not CD3delta, haploinsufficiency differentially impairs gammadelta versus alphabeta surface TCR expression. BMC Immunol (2013) 14:3 10.1186/1471-2172-14-3 23336327 PMC3585704

[28] Kazen AR Adams EJ. Evolution of the V, D, and J gene segments used in the primate gammadelta T-cell receptor reveals a dichotomy of conservation and diversity. Proc Natl Acad Sci U S A (2011) 108(29):E332–40. 10.1073/pnas.1105105108 21730193 PMC3141992

[29] Borst J Wicherink A Van Dongen JJ De Vries E Comans-Bitter WM Wassenaar F Non-random expression of T cell receptor gamma and delta variable gene segments in functional T lymphocyte clones from human peripheral blood. Eur J Immunol (1989) 19(9):1559–68. 10.1002/eji.1830190907 2529123

[30] Morita CT Mariuzza RA Brenner MB Antigen recognition by human gamma delta T cells: pattern recognition by the adaptive immune system. Springer Semin Immunopathol (2000) 22(3):191–217. 10.1007/s002810000042 11116953

[31] Dimova T Brouwer M Gosselin F Tassignon J Leo O Donner C Effector Vgamma9Vdelta2 T cells dominate the human fetal gammadelta T-cell repertoire. Proc Natl Acad Sci U S A (2015) 112(6):E556–65. 10.1073/pnas.1412058112 25617367 PMC4330771

[32] Ravens S Schultze-Florey C Raha S Sandrock I Drenker M Oberdorfer L Human gammadelta T cells are quickly reconstituted after stem-cell transplantation and show adaptive clonal expansion in response to viral infection. Nat Immunol (2017) 18(4):393–401. 10.1038/ni.3686 28218745

[33] Pauza CD Cairo C. Evolution and function of the TCR Vgamma9 chain repertoire: it’s good to be public. Cell Immunol (2015) 296(1):22–30. 10.1016/j.cellimm.2015.02.010 25769734 PMC4466227

[34] Pont F Familiades J Déjean S Fruchon S Cendron D Poupot M The gene expression profile of phosphoantigen-specific human γδ T lymphocytes is a blend of αβ T-cell and NK-cell signatures. Eur J Immunol (2012) 42(1):228–40. 10.1002/eji.201141870 21968650

[35] Shen Y Zhou D Qiu L Lai X Simon M Shen L Adaptive immune response of Vgamma2Vdelta2+ T cells during mycobacterial infections. Science (2002) 295(5563):2255–8. 10.1126/science.1068819 11910108 PMC2872146

[36] Brandes M Willimann K Moser B. Professional antigen-presentation function by human gammadelta T cells. Science (2005) 309(5732):264–8. 10.1126/science.1110267 15933162

[37] Allison TJ Winter CC Fournie JJ Bonneville M Garboczi DN. Structure of a human gammadelta T-cell antigen receptor. Nature (2001) 411(6839):820–4. 10.1038/35081115 11459064

[38] Fisch P Malkovsky M Kovats S Sturm E Braakman E Klein BS Recognition by human V gamma 9/V delta 2 T cells of a GroEL homolog on Daudi Burkitt’s lymphoma cells. Science (1990) 250(4985):1269–73. 10.1126/science.1978758 1978758

[39] Lang F Peyrat MA Constant P Davodeau F David-Ameline J Poquet Y Early activation of human V gamma 9V delta 2 T cell broad cytotoxicity and TNF production by nonpeptidic mycobacterial ligands. J Immunol (1995) 154(11):5986–94. 7751641

[40] Constant P Davodeau F Peyrat MA Poquet Y Puzo G Bonneville M Stimulation of human gamma delta T cells by nonpeptidic mycobacterial ligands. Science (1994) 264(5156):267–70. 10.1126/science.8146660 8146660

[41] Tanaka Y Morita CT Tanaka Y Nieves E Brenner MB Bloom BR. Natural and synthetic non-peptide antigens recognized by human gamma delta T cells. Nature (1995) 375(6527):155–8. 10.1038/375155a0 7753173

[42] Karunakaran MM Herrmann T The Vgamma9Vdelta2 T cell antigen receptor and butyrophilin-3 A1: models of interaction, the possibility of co-evolution, and the case of dendritic epidermal T cells. Front Immunol (2014) 5:648 10.3389/fimmu.2014.00648 25566259 PMC4271611

[43] Dieli F Poccia F Lipp M Sireci G Caccamo N Di Sano C Differentiation of effector/memory Vdelta2 T cells and migratory routes in lymph nodes or inflammatory sites. J Exp Med (2003) 198(3):391–7. 10.1084/jem.20030235 12900516 PMC2194087

[44] Ryan PL Sumaria N Holland CJ Bradford CM Izotova N Grandjean CL Heterogeneous yet stable Vdelta2(+) T-cell profiles define distinct cytotoxic effector potentials in healthy human individuals. Proc Natl Acad Sci U S A (2016) 113(50):14378–83. 10.1073/pnas.1611098113 27911793 PMC5167212

[45] Fisher JP Heuijerjans J Yan M Gustafsson K Anderson J Gammadelta T cells for cancer immunotherapy: a systematic review of clinical trials. Oncoimmunology (2014) 3(1):e27572 10.4161/onci.27572 24734216 PMC3984269

[46] Kunzmann V Bauer E Feurle J Weissinger F Tony HP Wilhelm M. Stimulation of gammadelta T cells by aminobisphosphonates and induction of antiplasma cell activity in multiple myeloma. Blood (2000) 96(2):384–92. 10887096

[47] Todaro M D’Asaro M Caccamo N Iovino F Francipane MG Meraviglia S Efficient killing of human colon cancer stem cells by gammadelta T lymphocytes. J Immunol (2009) 182(11):7287–96. 10.4049/jimmunol.0804288 19454726

[48] Schilbach KE Geiselhart A Wessels JT Niethammer D Handgretinger R. Human gammadelta T lymphocytes exert natural and IL-2-induced cytotoxicity to neuroblastoma cells. J Immunother (2000) 23(5):536–48. 10.1097/00002371-200009000-00004 11001547

[49] Liu Z Guo BL Gehrs BC Nan L Lopez RD. Ex vivo expanded human Vgamma9Vdelta2+ gammadelta-T cells mediate innate antitumor activity against human prostate cancer cells in vitro. J Urol (2005) 173(5):1552–6. 10.1097/01.ju.0000154355.45816.0b 15821484

[50] Viey E Fromont G Escudier B Morel Y Da Rocha S Chouaib S Phosphostim-activated gamma delta T cells kill autologous metastatic renal cell carcinoma. J Immunol (2005) 174(3):1338–47. 10.4049/jimmunol.174.3.1338 15661891

[51] Corvaisier M Moreau-Aubry A Diez E Bennouna J Mosnier JF Scotet E V gamma 9V delta 2 T cell response to colon carcinoma cells. J Immunol (2005) 175(8):5481–8. 10.4049/jimmunol.175.8.5481 16210656

[52] Kong Y Cao W Xi X Ma C Cui L He W. The NKG2D ligand ULBP4 binds to TCRgamma9/delta2 and induces cytotoxicity to tumor cells through both TCRgammadelta and NKG2D. Blood (2009) 114(2):310–7. 10.1182/blood-2008-12-196287 19436053

[53] Toutirais O Cabillic F Le Friec G Salot S Loyer P Le Gallo M DNAX accessory molecule-1 (CD226) promotes human hepatocellular carcinoma cell lysis by Vγ9Vδ2 T cells. Eur J Immunol (2009) 39(5):1361–8. 10.1002/eji.200838409 19404979

[54] Aggarwal R Lu J Kanji S Das M Joseph M Lustberg MB Human Vgamma2Vdelta2 T cells limit breast cancer growth by modulating cell survival-, apoptosis-related molecules and microenvironment in tumors. Int J Cancer (2013) 133(9):2133–44. 10.1002/ijc.28217 23595559 PMC3939063

[55] Wrobel P Shojaei H Schittek B Gieseler F Wollenberg B Kalthoff H Lysis of a broad range of epithelial tumour cells by human gamma delta T cells: involvement of NKG2D ligands and T-cell receptor-versus NKG2D-dependent recognition. Scand J Immunol (2007) 66(2–3):320–8. 10.1111/j.1365-3083.2007.01963.x 17635809

[56] Malkovska V Cigel FK Armstrong N Storer BE Hong R. Antilymphoma activity of human gamma delta T-cells in mice with severe combined immune deficiency. Cancer Res (1992) 52(20):5610–6. 1394184

[57] Wang L Kamath A Das H Li L Bukowski JF. Antibacterial effect of human V gamma 2V delta 2 T cells in vivo. J Clin Invest (2001) 108(9):1349–57. 10.1172/JCI13584 11696580 PMC209444

[58] Kabelitz D Wesch D Pitters E Zoller M. Characterization of tumor reactivity of human V gamma 9V delta 2 gamma delta T cells in vitro and in SCID mice in vivo. J Immunol (2004) 173(11):6767–76. 10.4049/jimmunol.173.11.6767 15557170

[59] Casetti R Perretta G Taglioni A Mattei M Colizzi V Dieli F Drug-induced expansion and differentiation of V gamma 9V delta 2 T cells in vivo: the role of exogenous IL-2. J Immunol (2005) 175(3):1593–8. 10.4049/jimmunol.175.3.1593 16034098

[60] Simoni D Gebbia N Invidiata FP Eleopra M Marchetti P Rondanin R Design, synthesis, and biological evaluation of novel aminobisphosphonates possessing an in vivo antitumor activity through a gammadelta-T lymphocytes-mediated activation mechanism. J Med Chem (2008) 51(21):6800–7. 10.1021/jm801003y 18937434

[61] Beck BH Kim HG Kim H Samuel S Liu Z Shrestha R Adoptively transferred ex vivo expanded gammadelta-T cells mediate in vivo antitumor activity in preclinical mouse models of breast cancer. Breast Cancer Res Treat (2010) 122(1):135–44. 10.1007/s10549-009-0527-6 19763820 PMC2883655

[62] D’Asaro M La Mendola C Di Liberto D Orlando V Todaro M Spina M V gamma 9V delta 2 T lymphocytes efficiently recognize and kill zoledronate-sensitized, imatinib-sensitive, and imatinib-resistant chronic myelogenous leukemia cells. J Immunol (2010) 184(6):3260–8. 10.4049/jimmunol.0903454 20154204

[63] Capietto AH Martinet L Fournie JJ Stimulated gammadelta T cells increase the in vivo efficacy of trastuzumab in HER-2+ breast cancer. J Immunol (2011) 187(2):1031–8. 10.4049/jimmunol.1100681 21670311

[64] Siegers GM Felizardo TC Mathieson AM Kosaka Y Wang X-H Medin JA Anti-leukemia activity of in vitro-expanded human gamma delta T cells in a xenogeneic Ph(+) leukemia model. PLoS One (2011) 6(2):e16700 10.1371/journal.pone.0016700 21304898 PMC3033392

[65] Santolaria T Robard M Léger A Catros V Bonneville M Scotet E Repeated systemic administrations of both aminobisphosphonates and human Vγ9Vδ2 T cells efficiently control tumor development in vivo. J Immunol (2013) 191(4):1993–2000. 10.4049/jimmunol.1300255 23836057

[66] Sun L Li Y Jiang Z Zhang J Li H Li B Vgamma9Vdelta2 T cells and zoledronate mediate antitumor activity in an orthotopic mouse model of human chondrosarcoma. Tumour Biol (2016) 37(6):7333–44. 10.1007/s13277-015-4615-4 26676633

[67] Nada MH Wang H Workalemahu G Tanaka Y Morita CT Enhancing adoptive cancer immunotherapy with Vgamma2Vdelta2 T cells through pulse zoledronate stimulation. J Immunother Cancer (2017) 5:9 10.1186/s40425-017-0209-6 28239463 PMC5319075

[68] Zysk A DeNichilo MO Panagopoulos V Zinonos I Liapis V Hay S Adoptive transfer of ex vivo expanded Vγ9Vδ2 T cells in combination with zoledronic acid inhibits cancer growth and limits osteolysis in a murine model of osteolytic breast cancer. Cancer Lett (2017) 386:141–50. 10.1016/j.canlet.2016.11.013 27865798 PMC5568037

[69] Pennington DJ Vermijlen D Wise EL Clarke SL Tigelaar RE Hayday AC. The integration of conventional and unconventional T cells that characterizes cell-mediated responses. Adv Immunol (2005) 87:27–59. 10.1016/S0065-2776(05)87002-6 16102571

[70] Lo Presti E Dieli F Meraviglia S Tumor-infiltrating gammadelta T lymphocytes: pathogenic role, clinical significance, and differential programing in the tumor microenvironment. Front Immunol (2014) 5:607 10.3389/fimmu.2014.00607 25505472 PMC4241840

[71] Meraviglia S Eberl M Vermijlen D Todaro M Buccheri S Cicero G In vivo manipulation of Vgamma9Vdelta2 T cells with zoledronate and low-dose interleukin-2 for immunotherapy of advanced breast cancer patients. Clin Exp Immunol (2010) 161(2):290–7. 10.1111/j.1365-2249.2010.04167.x 20491785 PMC2909411

[72] Kunzmann V Smetak M Kimmel B Weigang-Koehler K Goebeler M Birkmann J Tumor-promoting versus tumor-antagonizing roles of gammadelta T cells in cancer immunotherapy: results from a prospective phase I/II trial. J Immunother (2012) 35(2):205–13. 10.1097/CJI.0b013e318245bb1e 22306909

[73] Bennouna J Levy V Sicard H Senellart H Audrain M Hiret S Phase I study of bromohydrin pyrophosphate (BrHPP, IPH 1101), a Vgamma9Vdelta2 T lymphocyte agonist in patients with solid tumors. Cancer Immunol Immunother (2010) 59(10):1521–30. 10.1007/s00262-010-0879-0 20563721 PMC11030967

[74] Lang JM Kaikobad MR Wallace M Staab MJ Horvath DL Wilding G Pilot trial of interleukin-2 and zoledronic acid to augment gammadelta T cells as treatment for patients with refractory renal cell carcinoma. Cancer Immunol Immunother (2011) 60(10):1447–60. 10.1007/s00262-011-1049-8 21647691 PMC3177972

[75] Bennouna J Bompas E Neidhardt EM Rolland F Philip I Galea C Phase-I study of Innacell gammadelta, an autologous cell-therapy product highly enriched in gamma9delta2 T lymphocytes, in combination with IL-2, in patients with metastatic renal cell carcinoma. Cancer Immunol Immunother (2008) 57(11):1599–609. 10.1007/s00262-008-0491-8 18301889 PMC11030608

[76] Sakamoto M Nakajima J Murakawa T Fukami T Yoshida Y Murayama T Adoptive immunotherapy for advanced non-small cell lung cancer using zoledronate-expanded gammadeltaTcells: a phase I clinical study. J Immunother (2011) 34(2):202–11. 10.1097/CJI.0b013e318207ecfb 21304399

[77] Noguchi A Kaneko T Kamigaki T Fujimoto K Ozawa M Saito M Zoledronate-activated Vgamma9gammadelta T cell-based immunotherapy is feasible and restores the impairment of gammadelta T cells in patients with solid tumors. Cytotherapy (2011) 13(1):92–7. 10.3109/14653249.2010.515581 20831354

[78] Kobayashi H Tanaka Y Yagi J Osaka Y Nakazawa H Uchiyama T Safety profile and anti-tumor effects of adoptive immunotherapy using gamma-delta T cells against advanced renal cell carcinoma: a pilot study. Cancer Immunol Immunother (2007) 56(4):469–76. 10.1007/s00262-006-0199-6 16850345 PMC11030814

[79] Kobayashi H Tanaka Y Yagi J Minato N Tanabe K Phase I/II study of adoptive transfer of gammadelta T cells in combination with zoledronic acid and IL-2 to patients with advanced renal cell carcinoma. Cancer Immunol Immunother (2011) 60(8):1075–84. 10.1007/s00262-011-1021-7 21519826 PMC11029699

[80] Abe Y Muto M Nieda M Nakagawa Y Nicol A Kaneko T Clinical and immunological evaluation of zoledronate-activated Vgamma9gammadelta T-cell-based immunotherapy for patients with multiple myeloma. Exp Hematol (2009) 37(8):956–68. 10.1016/j.exphem.2009.04.008 19409955

[81] Nakajima J Murakawa T Fukami T Goto S Kaneko T Yoshida Y A phase I study of adoptive immunotherapy for recurrent non-small-cell lung cancer patients with autologous gammadelta T cells. Eur J Cardiothorac Surg (2010) 37(5):1191–7. 10.1016/j.ejcts.2009.11.051 20137969

[82] Wilhelm M Smetak M Schaefer-Eckart K Kimmel B Birkmann J Einsele H Successful adoptive transfer and in vivo expansion of haploidentical gammadelta T cells. J Transl Med (2014) 12:45 10.1186/1479-5876-12-45 24528541 PMC3926263

[83] Izumi T Kondo M Takahashi T Fujieda N Kondo A Tamura N Ex vivo characterization of gammadelta T-cell repertoire in patients after adoptive transfer of Vgamma9Vdelta2 T cells expressing the interleukin-2 receptor beta-chain and the common gamma-chain. Cytotherapy (2013) 15(4):481–91. 10.1016/j.jcyt.2012.12.004 23391461

[84] Nicol AJ Tokuyama H Mattarollo SR Hagi T Suzuki K Yokokawa K Clinical evaluation of autologous gamma delta T cell-based immunotherapy for metastatic solid tumours. Br J Cancer (2011) 105(6):778–86. 10.1038/bjc.2011.293 21847128 PMC3171009

[85] Wada I Matsushita H Noji S Mori K Yamashita H Nomura S Intraperitoneal injection of in vitro expanded Vgamma9Vdelta2 T cells together with zoledronate for the treatment of malignant ascites due to gastric cancer. Cancer Med (2014) 3(2):362–75. 10.1002/cam4.196 24515916 PMC3987085

[86] Cui J Wang N Zhao H Jin H Wang G Niu C Combination of radiofrequency ablation and sequential cellular immunotherapy improves progression-free survival for patients with hepatocellular carcinoma. Int J Cancer (2014) 134(2):342–51. 10.1002/ijc.28372 23825037

[87] Lo Presti E Pizzolato G Gulotta E Cocorullo G Gulotta G Dieli F Current advances in gammadelta T cell-based tumor immunotherapy. Front Immunol (2017) 8:1401 10.3389/fimmu.2017.01401 29163482 PMC5663908

[88] Kobayashi H Tanaka Y Gammadelta T cell immunotherapy—a review. Pharmaceuticals (2015) 8(1):40–61. 10.3390/ph8010040 25686210 PMC4381201

[89] Laurent G de Micheaux SL Solal-Celigny P Soubeyran P Delwail V Ghesquières H Phase I/II study of IPH1101, γσ T cell agonist, combined with rituximab, in low grade follicular lymphoma patients. Blood (2009) 114(22):1649.

[90] Aoki T Matsushita H Hoshikawa M Hasegawa K Kokudo N Kakimi K Adjuvant combination therapy with gemcitabine and autologous gammadelta T-cell transfer in patients with curatively resected pancreatic cancer. Cytotherapy (2017) 19(4):473–85. 10.1016/j.jcyt.2017.01.002 28188072

[91] Siegers GM Swamy M Fernandez-Malave E Minguet S Rathmann S Guardo AC Different composition of the human and the mouse gammadelta T cell receptor explains different phenotypes of CD3gamma and CD3delta immunodeficiencies. J Exp Med (2007) 204(11):2537–44. 10.1084/jem.20070782 17923503 PMC2118495

[92] Su C Jakobsen I Gu X Nei M. Diversity and evolution of T-cell receptor variable region genes in mammals and birds. Immunogenetics (1999) 50(5–6):301–8. 10.1007/s002510050606 10630294

[93] Vantourout P Hayday A Six-of-the-best: unique contributions of gammadelta T cells to immunology. Nat Rev Immunol (2013) 13(2):88–100. 10.1038/nri3384 23348415 PMC3951794

[94] Brandes M Willimann K Lang AB Nam K-H Jin C Brenner MB Flexible migration program regulates γδ T-cell involvement in humoral immunity. Blood (2003) 102(10):3693–701. 10.1182/blood-2003-04-1016 12881309

[95] Khan MW Eberl M Moser B Potential use of [gammadelta] T cell-based vaccines in cancer immunotherapy. Front Immunol (2014) 5:512 10.3389/fimmu.2014.00512 25374569 PMC4204533

[96] Paul S Lal G Regulatory and effector functions of gamma–delta (γδ) T cells and their therapeutic potential in adoptive cellular therapy for cancer. Int J Cancer (2016) 139(5):976–85. 10.1002/ijc.30109 27012367

[97] Marcu-Malina V Heijhuurs S van Buuren M Hartkamp L Strand S Sebestyen Z Redirecting alphabeta T cells against cancer cells by transfer of a broadly tumor-reactive gammadeltaT-cell receptor. Blood (2011) 118(1):50–9. 10.1182/blood-2010-12-325993 21566093

[98] Ribeiro ST Ribot JC Silva-Santos B Five layers of receptor signaling in gammadelta T-cell differentiation and activation. Front Immunol (2015) 6:15 10.3389/fimmu.2015.00015 25674089 PMC4306313

[99] Gruenbacher G Nussbaumer O Gander H Steiner B Leonhartsberger N Thurnher M Stress-related and homeostatic cytokines regulate Vgamma9Vdelta2 T-cell surveillance of mevalonate metabolism. Oncoimmunology (2014) 3(8):e953410 10.4161/21624011.2014.953410 25960933 PMC4368140

[100] Hanahan D Weinberg RA Hallmarks of cancer: the next generation. Cell (2011) 144(5):646–74. 10.1016/j.cell.2011.02.013 21376230

[101] Warburg O Posener K Negelein E Über den Stoffwechsel der Carcinomzelle. Biochem Z (1924) 152:309–44.

[102] Renner K Singer K Koehl GE Geissler EK Peter K Siska PJ Metabolic hallmarks of tumor and immune cells in the tumor microenvironment. Front Immunol (2017) 8:248. 10.3389/fimmu.2017.00248 28337200 PMC5340776

[103] Altincicek B Moll J Campos N Foerster G Beck E Hoeffler J-F Cutting edge: human gamma delta T cells are activated by intermediates of the 2-C-methyl-D-erythritol 4-phosphate pathway of isoprenoid biosynthesis. J Immunol (2001) 166(6):3655–8. 10.4049/jimmunol.166.6.3655 11238603

[104] Coscia M Vitale C Peola S Foglietta M Rigoni M Griggio V Dysfunctional Vgamma9Vdelta2 T cells are negative prognosticators and markers of dysregulated mevalonate pathway activity in chronic lymphocytic leukemia cells. Blood (2012) 120(16):3271–9. 10.1182/blood-2012-03-417519 22932792

[105] Gober HJ Kistowska M Angman L Jeno P Mori L De Libero G. Human T cell receptor gammadelta cells recognize endogenous mevalonate metabolites in tumor cells. J Exp Med (2003) 197(2):163–8. 10.1084/jem.20021500 12538656 PMC2193814

[106] Gruenbacher G Thurnher M. Mevalonate metabolism governs cancer immune surveillance. Oncoimmunology (2017) 6(10):e1342917. 10.1080/2162402X.2017.1342917 29123952 PMC5665080

[107] Harwood HJ Jr Alvarez IM Noyes WD Stacpoole PW. In vivo regulation of human leukocyte 3-hydroxy-3-methylglutaryl coenzyme A reductase: increased enzyme protein concentration and catalytic efficiency in human leukemia and lymphoma. J Lipid Res (1991) 32(8):1237–52. 1770307

[108] Morita CT Jin C Sarikonda G Wang H. Nonpeptide antigens, presentation mechanisms, and immunological memory of human Vgamma2Vdelta2 T cells: discriminating friend from foe through the recognition of prenyl pyrophosphate antigens. Immunol Rev (2007) 215:59–76. 10.1111/j.1600-065X.2006.00479.x 17291279

[109] De Libero G Sentinel function of broadly reactive human gamma delta T cells. Immunol Today (1997) 18(1):22–6. 10.1016/S0167-5699(97)80010-2 9018970

[110] Moulin M Alguacil J Gu S Mehtougui A Adams EJ Peyrottes S Vgamma9Vdelta2 T cell activation by strongly agonistic nucleotidic phosphoantigens. Cell Mol Life Sci (2017) 74(23):4353–67. 10.1007/s00018-017-2583-0 28669030 PMC11107656

[111] Kunzmann V Bauer E Wilhelm M Gamma/delta T-cell stimulation by pamidronate. N Engl J Med (1999) 340(9):737–8. 10.1056/NEJM199903043400914 10068336

[112] Wiemer DF Wiemer AJ Opportunities and challenges in development of phosphoantigens as Vgamma9Vdelta2 T cell agonists. Biochem Pharmacol (2014) 89(3):301–12. 10.1016/j.bcp.2014.03.009 24680696

[113] Jauhiainen M Monkkonen H Raikkonen J Monkkonen J Auriola S. Analysis of endogenous ATP analogs and mevalonate pathway metabolites in cancer cell cultures using liquid chromatography-electrospray ionization mass spectrometry. J Chromatogr (2009) 877(27):2967–75. 10.1016/j.jchromb.2009.07.010 19665949

[114] Gundermann S Klinker E Kimmel B Flierl U Wilhelm M Einsele H A comprehensive analysis of primary acute myeloid leukemia identifies biomarkers predicting susceptibility to human allogeneic Vgamma9Vdelta2 T cells. J Immunother (2014) 37(6):321–30. 10.1097/CJI.0000000000000043 24911793

[115] Idrees AS Sugie T Inoue C Murata-Hirai K Okamura H Morita CT Comparison of gammadelta T cell responses and farnesyl diphosphate synthase inhibition in tumor cells pretreated with zoledronic acid. Cancer Sci (2013) 104(5):536–42. 10.1111/cas.12124 23387443 PMC3755363

[116] Van Acker HH Anguille S Willemen Y Smits EL Van Tendeloo VF. Bisphosphonates for cancer treatment: mechanisms of action and lessons from clinical trials. Pharmacol Ther (2016) 158:24–40. 10.1016/j.pharmthera.2015.11.008 26617219

[117] Espinosa E Belmant C Pont F Luciani B Poupot R Romagne F Chemical synthesis and biological activity of bromohydrin pyrophosphate, a potent stimulator of human gamma delta T cells. J Biol Chem (2001) 276(21):18337–44. 10.1074/jbc.M100495200 11279081

[118] Lafont V Sanchez F Laprevotte E Michaud HA Gros L Eliaou JF Plasticity of gammadelta T cells: impact on the anti-tumor response. Front Immunol (2014) 5:622 10.3389/fimmu.2014.00622 25538706 PMC4259167

[119] Wang H Fang Z Morita CT. Vgamma2Vdelta2 T cell receptor recognition of prenyl pyrophosphates is dependent on all CDRs. J Immunol (2010) 184(11):6209–22. 10.4049/jimmunol.1000231 20483784 PMC3069129

[120] Rakasz E MacDougall AV Zayas MT Helgelund JL Ruckward TJ Hatfield G Gammadelta T cell receptor repertoire in blood and colonic mucosa of rhesus macaques. J Med Primatol (2000) 29(6):387–96. 10.1111/j.1600-0684.2000.290602.x 11168829

[121] Karunakaran MM Gobel TW Starick L Walter L Herrmann T Vgamma9 and Vdelta2 T cell antigen receptor genes and butyrophilin 3 (BTN3) emerged with placental mammals and are concomitantly preserved in selected species like alpaca (Vicugna pacos). Immunogenetics (2014) 66(4):243–54. 10.1007/s00251-014-0763-8 24526346

[122] Bukowski JF Morita CT Tanaka Y Bloom BR Brenner MB Band H. V gamma 2V delta 2 TCR-dependent recognition of non-peptide antigens and Daudi cells analyzed by TCR gene transfer. J Immunol (1995) 154(3):998–1006. 7529807

[123] Xiang Z Liu Y Zheng J Liu M Lv A Gao Y Targeted activation of human Vgamma9Vdelta2-T cells controls epstein-barr virus-induced B cell lymphoproliferative disease. Cancer Cell (2014) 26(4):565–76. 10.1016/j.ccr.2014.07.026 25220446

[124] Fisher JP Flutter B Wesemann F Frosch J Rossig C Gustafsson K Effective combination treatment of GD2-expressing neuroblastoma and Ewing’s sarcoma using anti-GD2 ch14.18/CHO antibody with Vgamma9Vdelta2+ gammadeltaT cells. Oncoimmunology (2016) 5(1):e1025194 10.1080/2162402X.2015.1025194 26942051 PMC4760299

[125] Morgan GJ Davies FE Gregory WM Cocks K Bell SE Szubert AJ First-line treatment with zoledronic acid as compared with clodronic acid in multiple myeloma (MRC myeloma IX): a randomised controlled trial. Lancet (2010) 376(9757):1989–99. 10.1016/S0140-6736(10)62051-X 21131037 PMC3639680

[126] Dieli F Gebbia N Poccia F Caccamo N Montesano C Fulfaro F Induction of gammadelta T-lymphocyte effector functions by bisphosphonate zoledronic acid in cancer patients in vivo. Blood (2003) 102(6):2310–1. 10.1182/blood-2003-05-1655 12959943

[127] Lamb LS Jr Musk P Ye Z van Rhee F Geier SS Tong JJ Human gammadelta(+) T lymphocytes have in vitro graft vs leukemia activity in the absence of an allogeneic response. Bone Marrow Transplant (2001) 27(6):601–6. 10.1038/sj.bmt.1702830 11319589

[128] Rosenberg SA. IL-2: the first effective immunotherapy for human cancer. J Immunol (2014) 192(12):5451–8. 10.4049/jimmunol.1490019 24907378 PMC6293462

[129] Miyagawa F Tanaka Y Yamashita S Minato N. Essential requirement of antigen presentation by monocyte lineage cells for the activation of primary human gamma delta T cells by aminobisphosphonate antigen. J Immunol (2001) 166(9):5508–14. 10.4049/jimmunol.166.9.5508 11313389

[130] Roelofs AJ Jauhiainen M Monkkonen H Rogers MJ Monkkonen J Thompson K. Peripheral blood monocytes are responsible for gammadelta T cell activation induced by zoledronic acid through accumulation of IPP/DMAPP. Br J Haematol (2009) 144(2):245–50. 10.1111/j.1365-2141.2008.07435.x 19016713 PMC2659391

[131] Fowler DW Copier J Dalgleish AG Bodman-Smith MD Zoledronic acid renders human M1 and M2 macrophages susceptible to Vdelta2(+) gammadelta T cell cytotoxicity in a perforin-dependent manner. Cancer Immunol Immunother (2017) 66(9):1205–15. 10.1007/s00262-017-2011-1 28501938 PMC5579165

[132] Kalyan S Chandrasekaran V Quabius ES Lindhorst TK Kabelitz D Neutrophil uptake of nitrogen-bisphosphonates leads to the suppression of human peripheral blood gammadelta T cells. Cell Mol Life Sci (2014) 71(12):2335–46. 10.1007/s00018-013-1495-x 24162933 PMC11114071

[133] Rossini M Adami S Viapiana O Fracassi E Ortolani R Vella A Long-term effects of amino-bisphosphonates on circulating gammadelta T cells. Calcif Tissue Int (2012) 91(6):395–9. 10.1007/s00223-012-9647-9 23052225

[134] Workalemahu G Wang H Puan KJ Nada MH Kuzuyama T Jones BD Metabolic engineering of *Salmonella* vaccine bacteria to boost human Vgamma2Vdelta2 T cell immunity. J Immunol (2014) 193(2):708–21. 10.4049/jimmunol.1302746 24943221 PMC4241231

[135] Jarry U Chauvin C Joalland N Leger A Minault S Robard M Stereotaxic administrations of allogeneic human Vgamma9Vdelta2 T cells efficiently control the development of human glioblastoma brain tumors. Oncoimmunology (2016) 5(6):e1168554 10.1080/2162402X.2016.1168554 27471644 PMC4938356

[136] Kennel KA Drake MT. Adverse effects of bisphosphonates: implications for osteoporosis management. Mayo Clin Proc (2009) 84(7):632–8. 10.1016/S0025-6196(11)60752-0 19567717 PMC2704135

[137] Hsiao CH Lin X Barney RJ Shippy RR Li J Vinogradova O Synthesis of a phosphoantigen prodrug that potently activates Vgamma9Vdelta2 T-lymphocytes. Chem Biol (2014) 21(8):945–54. 10.1016/j.chembiol.2014.06.006 25065532

[138] Tanaka Y Iwasaki M Murata-Hirai K Matsumoto K Hayashi K Okamura H Anti-tumor activity and immunotherapeutic potential of a bisphosphonate prodrug. Sci Rep (2017) 7(1):5987. 10.1038/s41598-017-05553-0 28729550 PMC5519590

[139] Matsumoto K Hayashi K Murata-Hirai K Iwasaki M Okamura H Minato N Targeting cancer cells with a bisphosphonate prodrug. ChemMedChem (2016) 11(24):2656–63. 10.1002/cmdc.201600465 27786425 PMC5605902

[140] Kilcollins AM Li J Hsiao C-HC Wiemer AJ HMBPP analog prodrugs bypass energy-dependent uptake to promote efficient BTN3A1-mediated malignant cell lysis by Vγ9Vδ2 T lymphocyte effectors. J Immunol (2016) 197(2):419–28. 10.4049/jimmunol.1501833 27271567 PMC4935553

[141] Hodgins NO Wang JT Al-Jamal KT Nano-technology based carriers for nitrogen-containing bisphosphonates delivery as sensitisers of gammadelta T cells for anticancer immunotherapy. Adv Drug Deliv Rev (2017) 114:143–60. 10.1016/j.addr.2017.07.003 28694026

[142] Zhou X Gu Y Xiao H Kang N Xie Y Zhang G Combining Vgamma9Vdelta2 T cells with a lipophilic bisphosphonate efficiently kills activated hepatic stellate cells. Front Immunol (2017) 8:1381 10.3389/fimmu.2017.01381 29118758 PMC5661056

[143] Zhang Y Cao R Yin F Lin FY Wang H Krysiak K Lipophilic pyridinium bisphosphonates: potent gammadelta T cell stimulators. Angew Chem Int Ed Engl (2010) 49(6):1136–8. 10.1002/anie.200905933 20039246 PMC2819003

[144] Harly C Guillaume Y Nedellec S Peigne CM Monkkonen H Monkkonen J Key implication of CD277/butyrophilin-3 (BTN3A) in cellular stress sensing by a major human gammadelta T-cell subset. Blood (2012) 120(11):2269–79. 10.1182/blood-2012-05-430470 22767497 PMC3679641

[145] Vavassori S Kumar A Wan GS Ramanjaneyulu GS Cavallari M El Daker S Butyrophilin 3A1 binds phosphorylated antigens and stimulates human gammadelta T cells. Nat Immunol (2013) 14(9):908–16. 10.1038/ni.2665 23872678

[146] Compte E Pontarotti P Collette Y Lopez M Olive D. Frontline: characterization of BT3 molecules belonging to the B7 family expressed on immune cells. Eur J Immunol (2004) 34(8):2089–99. 10.1002/eji.200425227 15259006

[147] Nguyen K Li J Puthenveetil R Lin X Poe MM Hsiao CC The butyrophilin 3A1 intracellular domain undergoes a conformational change involving the juxtamembrane region. FASEB J (2017) 31(11):4697–706. 10.1096/fj.201601370RR 28705810 PMC5636706

[148] Sandstrom A Peigne CM Leger A Crooks JE Konczak F Gesnel MC The intracellular B30.2 domain of butyrophilin 3A1 binds phosphoantigens to mediate activation of human Vgamma9Vdelta2 T cells. Immunity (2014) 40(4):490–500. 10.1016/j.immuni.2014.03.003 24703779 PMC4028361

[149] Wang H Henry O Distefano MD Wang YC Raikkonen J Monkkonen J Butyrophilin 3A1 plays an essential role in prenyl pyrophosphate stimulation of human Vgamma2Vdelta2 T cells. J Immunol (2013) 191(3):1029–42. 10.4049/jimmunol.1300658 23833237 PMC3884521

[150] Wang H Morita CT Sensor function for butyrophilin 3A1 in prenyl pyrophosphate stimulation of human Vgamma2Vdelta2 T cells. J Immunol (2015) 195(10):4583–94. 10.4049/jimmunol.1500314 26475929 PMC4848273

[151] Gu S Sachleben JR Boughter CT Nawrocka WI Borowska MT Tarrasch JT Phosphoantigen-induced conformational change of butyrophilin 3A1 (BTN3A1) and its implication on Vgamma9Vdelta2 T cell activation. Proc Natl Acad Sci U S A (2017) 114(35):E7311–20. 10.1073/pnas.1707547114 28807997 PMC5584448

[152] Sebestyen Z Scheper W Vyborova A Gu S Rychnavska Z Schiffler M RhoB mediates phosphoantigen recognition by Vgamma9Vdelta2 T cell receptor. Cell Rep (2016) 15(9):1973–85. 10.1016/j.celrep.2016.04.081 27210746 PMC5035041

[153] Castella B Kopecka J Sciancalepore P Mandili G Foglietta M Mitro N The ATP-binding cassette transporter A1 regulates phosphoantigen release and Vgamma9Vdelta2 T cell activation by dendritic cells. Nat Commun (2017) 8:15663 10.1038/ncomms15663 28580927 PMC5465356

[154] Messal N Mamessier E Sylvain A Celis-Gutierrez J Thibult ML Chetaille B Differential role for CD277 as a co-regulator of the immune signal in T and NK cells. Eur J Immunol (2011) 41(12):3443–54. 10.1002/eji.201141404 21918970

[155] Decaup E Duault C Bezombes C Poupot M Savina A Olive D Phosphoantigens and butyrophilin 3A1 induce similar intracellular activation signaling in human TCRVgamma9(+) gammadelta T lymphocytes. Immunol Lett (2014) 161(1):133–7. 10.1016/j.imlet.2014.05.011 24925024

[156] Starick L Riano F Karunakaran MM Kunzmann V Li J Kreiss M Butyrophilin 3A (BTN3A, CD277)-specific antibody 20.1 differentially activates Vgamma9Vdelta2 TCR clonotypes and interferes with phosphoantigen activation. Eur J Immunol (2017) 47(6):982–92. 10.1002/eji.201646818 28386905

[157] Benyamine A Le Roy A Mamessier E Gertner-Dardenne J Castanier C Orlanducci F BTN3A molecules considerably improve Vγ9Vδ2T cells-based immunotherapy in acute myeloid leukemia. Oncoimmunology (2016) 5(10):e1146843. 10.1080/2162402X.2016.1146843 27853633 PMC5087298

[158] Hoeres T Guenther R Smetak M Holzmann E Birkmann J Wilhelm M Murine anti-CD277 antibody increases the cytotoxic effect of gammadelta T-cells against human primary leukemia cells in vitro. In: Wilhelm M, editor. Paracelsus Science Get Together 2015; 03.07.2015; Nuremberg. Nuremberg: Forschungsbüro der PMU Nürnberg (2015). 66 p.

[159] Uhlen M Fagerberg L Hallstrom BM Lindskog C Oksvold P Mardinoglu A Proteomics. Tissue-based map of the human proteome. Science (2015) 347(6220):1260419. 10.1126/science.1260419 25613900

[160] Kankotia S Stacpoole PW. Dichloroacetate and cancer: new home for an orphan drug? Biochim Biophys Acta (2014) 1846(2):617–29. 10.1016/j.bbcan.2014.08.005 25157892

[161] Hoeres T Holzmann E Smetak M Birkmann J Wilhelm M Dichloroacetate inhibits aerobic glycolysis and proliferation of certain leukemia cell lines and can enhance anti-tumor effects of gamma-delta T-cells. In: Rasp G, editor. Paracelsus Science Get Together 2016; 24.06.2016; Salzburg. Salzburg: Forschungsbüro der PMU-Salzburg (2016). 107 p.

[162] Gonnermann D Oberg H-H Kellner C Peipp M Sebens S Kabelitz D Resistance of cyclooxygenase-2 expressing pancreatic ductal adenocarcinoma cells against γδ T cell cytotoxicity. Oncoimmunology (2015) 4(3):e988460. 10.4161/2162402X.2014.988460 25949900 PMC4404835

[163] Zhao H Bo C Kang Y Li H. What else can CD39 tell us? Front Immunol (2017) 8:727. 10.3389/fimmu.2017.00727 28690614 PMC5479880

[164] Gruenbacher G Gander H Rahm A Idzko M Nussbaumer O Thurnher M Ecto-ATPase CD39 inactivates isoprenoid-derived Vgamma9Vdelta2 T cell phosphoantigens. Cell Rep (2016) 16(2):444–56. 10.1016/j.celrep.2016.06.009 27346340

[165] Egler RA Ahuja SP Matloub Y L-asparaginase in the treatment of patients with acute lymphoblastic leukemia. J Pharmacol Pharmacother (2016) 7(2):62–71. 10.4103/0976-500X.184769 27440950 PMC4936081

[166] Chang CH Pearce EL. Emerging concepts of T cell metabolism as a target of immunotherapy. Nat Immunol (2016) 17(4):364–8. 10.1038/ni.3415 27002844 PMC4990080

[167] Bauer S Groh V Wu J Steinle A Phillips JH Lanier LL Activation of NK cells and T cells by NKG2D, a receptor for stress-inducible MICA. Science (1999) 285(5428):727–9. 10.1126/science.285.5428.727 10426993

[168] Diefenbach A Jamieson AM Liu SD Shastri N Raulet DH. Ligands for the murine NKG2D receptor: expression by tumor cells and activation of NK cells and macrophages. Nat Immunol (2000) 1(2):119–26. 10.1038/77793 11248803

[169] Groh V Rhinehart R Randolph-Habecker J Topp MS Riddell SR Spies T. Costimulation of CD8alphabeta T cells by NKG2D via engagement by MIC induced on virus-infected cells. Nat Immunol (2001) 2(3):255–60. 10.1038/85321 11224526

[170] Garrity D Call ME Feng J Wucherpfennig KW. The activating NKG2D receptor assembles in the membrane with two signaling dimers into a hexameric structure. Proc Natl Acad Sci U S A (2005) 102(21):7641–6. 10.1073/pnas.0502439102 15894612 PMC1140444

[171] Eagle RA Trowsdale J. Promiscuity and the single receptor: NKG2D. Nat Rev Immunol (2007) 7:737. 10.1038/nri2144 17673918

[172] Gasser S Orsulic S Brown EJ Raulet DH. The DNA damage pathway regulates innate immune system ligands of the NKG2D receptor. Nature (2005) 436(7054):1186–90. 10.1038/nature03884 15995699 PMC1352168

[173] Raulet DH. Roles of the NKG2D immunoreceptor and its ligands. Nat Rev Immunol (2003) 3(10):781–90. 10.1038/nri1199 14523385

[174] Obeidy P Sharland AF. NKG2D and its ligands. Int J Biochem Cell Biol (2009) 41(12):2364–7. 10.1016/j.biocel.2009.07.005 19631280

[175] Rincon-Orozco B Kunzmann V Wrobel P Kabelitz D Steinle A Herrmann T. Activation of V gamma 9V delta 2 T cells by NKG2D. J Immunol (2005) 175(4):2144–51. 10.4049/jimmunol.175.4.2144 16081780

[176] Shafi S Vantourout P Wallace G Antoun A Vaughan R Stanford M An NKG2D-mediated human lymphoid stress surveillance response with high interindividual variation. Sci Transl Med (2011) 3(113):113ra24. 10.1126/scitranslmed.3002922 22133594 PMC3966512

[177] Chen D Gyllensten U MICA polymorphism: biology and importance in cancer. Carcinogenesis (2014) 35(12):2633–42. 10.1093/carcin/bgu215 25330802

[178] Suresh PK. Membrane-bound versus soluble major histocompatibility complex class I-related chain A and major histocompatibility complex class I-related chain B differential expression: mechanisms of tumor eradication versus evasion and current drug development strategies. J Cancer Res Ther (2016) 12(4):1224–33. 10.4103/0973-1482.176169 28169232

[179] Marten A von Lilienfeld-Toal M Buchler MW Schmidt J. Soluble MIC is elevated in the serum of patients with pancreatic carcinoma diminishing gamma delta T cell cytotoxicity. Int J Cancer (2006) 119(10):2359–65. 10.1002/ijc.22186 16929491

[180] Lamb LS Jr Bowersock J Dasgupta A Gillespie GY Su Y Johnson A Engineered drug resistant gammadelta T cells kill glioblastoma cell lines during a chemotherapy challenge: a strategy for combining chemo- and immunotherapy. PLoS One (2013) 8(1):e51805 10.1371/journal.pone.0051805 23326319 PMC3543433

[181] Chitadze G Lettau M Luecke S Wang T Janssen O Furst D NKG2D- and T-cell receptor-dependent lysis of malignant glioma cell lines by human gammadelta T cells: modulation by temozolomide and A disintegrin and metalloproteases 10 and 17 inhibitors. Oncoimmunology (2016) 5(4):e1093276 10.1080/2162402X.2015.1093276 27141377 PMC4839372

[182] Flüh C Chitadze G Adamski V Hattermann K Synowitz M Kabelitz D NKG2D ligands in glioma stem-like cells: expression in situ and in vitro. Histochem Cell Biol (2018) 149(3):219–33. 10.1007/s00418-018-1633-5 29356965

[183] Hervieu A Rebe C Vegran F Chalmin F Bruchard M Vabres P Dacarbazine-mediated upregulation of NKG2D ligands on tumor cells activates NK and CD8 T cells and restrains melanoma growth. J Invest Dermatol (2013) 133(2):499–508. 10.1038/jid.2012.273 22951720

[184] Todaro M Orlando V Cicero G Caccamo N Meraviglia S Stassi G Chemotherapy sensitizes colon cancer initiating cells to Vgamma9Vdelta2 T cell-mediated cytotoxicity. PLoS One (2013) 8(6):e65145 10.1371/journal.pone.0065145 23762301 PMC3675136

[185] Mattarollo SR Kenna T Nieda M Nicol AJ. Chemotherapy and zoledronate sensitize solid tumour cells to Vgamma9Vdelta2 T cell cytotoxicity. Cancer Immunol Immunother (2007) 56(8):1285–97. 10.1007/s00262-007-0279-2 17265022 PMC11030464

[186] Guenther R Influence of Several Immunomodulatory Drugs and Receptor-Ligand-Systems on the γδ T Cell Effector Function against Leukemia. Nuremberg: Paracelsus Medical University (2015).

[187] Niu C Jin H Li M Zhu S Zhou L Jin F Low-dose bortezomib increases the expression of NKG2D and DNAM-1 ligands and enhances induced NK and gammadelta T cell-mediated lysis in multiple myeloma. Oncotarget (2017) 8(4):5954–64. 10.18632/oncotarget.13979 27992381 PMC5351604

[188] Bhat J Kabelitz D Gammadelta T cells and epigenetic drugs: a useful merger in cancer immunotherapy? Oncoimmunology (2015) 4(6):e1006088 10.1080/2162402x.2015.1006088 26155411 PMC4485805

[189] Suzuki T Terao S Acharya B Naoe M Yamamoto S Okamura H The antitumour effect of {gamma}{delta} T-cells is enhanced by valproic acid-induced up-regulation of NKG2D ligands. Anticancer Res (2010) 30(11):4509–13. 21115900

[190] Chavez-Blanco A De la Cruz-Hernandez E Dominguez GI Rodriguez-Cortez O Alatorre B Perez-Cardenas E Upregulation of NKG2D ligands and enhanced natural killer cell cytotoxicity by hydralazine and valproate. Int J Oncol (2011) 39(6):1491–9. 10.3892/ijo.2011.1144 21805029

[191] Satwani P Bavishi S Saha A Zhao F Ayello J van de Ven C Upregulation of NKG2D ligands in acute lymphoblastic leukemia and non-Hodgkin lymphoma cells by romidepsin and enhanced in vitro and in vivo natural killer cell cytotoxicity. Cytotherapy (2014) 16(10):1431–40. 10.1016/j.jcyt.2014.03.008 24856896

[192] Alsaab HO Sau S Alzhrani R Tatiparti K Bhise K Kashaw SK PD-1 and PD-L1 checkpoint signaling inhibition for cancer immunotherapy: mechanism, combinations, and clinical outcome. Front Pharmacol (2017) 8:561. 10.3389/fphar.2017.00561 28878676 PMC5572324

[193] Boussiotis VA Molecular and biochemical aspects of the PD-1 checkpoint pathway. N Engl J Med (2016) 375(18):1767–78. 10.1056/NEJMra1514296 27806234 PMC5575761

[194] Pedoeem A Azoulay-Alfaguter I Strazza M Silverman GJ Mor A Programmed death-1 pathway in cancer and autoimmunity. Clin Immunol (2014) 153(1):145–52. 10.1016/j.clim.2014.04.010 24780173

[195] Carey CD Gusenleitner D Lipschitz M Roemer MGM Stack EC Gjini E Topological analysis reveals a PD-L1-associated microenvironmental niche for Reed-Sternberg cells in Hodgkin lymphoma. Blood (2017) 130(22):2420–30. 10.1182/blood-2017-03-770719 28893733 PMC5766840

[196] Iwasaki M Tanaka Y Kobayashi H Murata-Hirai K Miyabe H Sugie T Expression and function of PD-1 in human gammadelta T cells that recognize phosphoantigens. Eur J Immunol (2011) 41(2):345–55. 10.1002/eji.201040959 21268005

[197] Paquin-Proulx D Barsotti NS Santos BA Marinho AK Kokron CM Carvalho KI Inversion of the Vdelta1 to Vdelta2 gammadelta T cell ratio in CVID is not restored by IVIg and is associated with immune activation and exhaustion. Medicine (Baltimore) (2016) 95(30):e4304 10.1097/MD.0000000000004304 27472706 PMC5265843

[198] Zumwalde NA Sharma A Xu X Ma S Schneider CL Romero-Masters JC Adoptively transferred Vgamma9Vdelta2 T cells show potent antitumor effects in a preclinical B cell lymphomagenesis model. JCI Insight (2017) 2(13):93179 10.1172/jci.insight.93179 28679955 PMC5499361

[199] Hsu H Boudova S Mvula G Divala TH Mungwira RG Harman C Prolonged PD1 expression on neonatal Vdelta2 lymphocytes dampens proinflammatory responses: role of epigenetic regulation. J Immunol (2016) 197(5):1884–92. 10.4049/jimmunol.1600284 27474072 PMC4992653

[200] Castella B Foglietta M Sciancalepore P Rigoni M Coscia M Griggio V Anergic bone marrow V gamma 9V delta 2 T cells as early and long-lasting markers of PD-1-targetable microenvironment-induced immune suppression in human myeloma. Oncoimmunology (2015) 4(11):e1047580 10.1080/2162402X.2015.1047580 26451323 PMC4589055

[201] Dondero A Pastorino F Della Chiesa M Corrias MV Morandi F Pistoia V PD-L1 expression in metastatic neuroblastoma as an additional mechanism for limiting immune surveillance. Oncoimmunology (2016) 5(1):e1064578 10.1080/2162402x.2015.1064578 26942080 PMC4760291

[202] Sakuishi K Apetoh L Sullivan JM Blazar BR Kuchroo VK Anderson AC. Targeting TIM-3 and PD-1 pathways to reverse T cell exhaustion and restore anti-tumor immunity. J Exp Med (2010) 207(10):2187–94. 10.1084/jem.20100643 20819927 PMC2947065

[203] Koyama S Akbay EA Li YY Herter-Sprie GS Buczkowski KA Richards WG Adaptive resistance to therapeutic PD-1 blockade is associated with upregulation of alternative immune checkpoints. Nat Commun (2016) 7:10501. 10.1038/ncomms10501 26883990 PMC4757784

[204] Watanabe N Gavrieli M Sedy JR Yang J Fallarino F Loftin SK BTLA is a lymphocyte inhibitory receptor with similarities to CTLA-4 and PD-1. Nat Immunol (2003) 4(7):670–9. 10.1038/ni944 12796776

[205] Gertner-Dardenne J Fauriat C Orlanducci F Thibult ML Pastor S Fitzgibbon J The co-receptor BTLA negatively regulates human Vgamma9Vdelta2 T-cell proliferation: a potential way of immune escape for lymphoma cells. Blood (2013) 122(6):922–31. 10.1182/blood-2012-11-464685 23692853

[206] Yang Z-Z Kim HJ Villasboas JC Chen Y-P Price-Troska T Jalali S Expression of LAG-3 defines exhaustion of intratumoral PD-1(+) T cells and correlates with poor outcome in follicular lymphoma. Oncotarget (2017) 8(37):61425–39. 10.18632/oncotarget.18251 28977875 PMC5617435

[207] Parry RV Chemnitz JM Frauwirth KA Lanfranco AR Braunstein I Kobayashi SV CTLA-4 and PD-1 receptors inhibit T-cell activation by distinct mechanisms. Mol Cell Biol (2005) 25(21):9543–53. 10.1128/MCB.25.21.9543-9553.2005 16227604 PMC1265804

[208] Miko E Szereday L Barakonyi A Jarkovich A Varga P Szekeres-Bartho J. Immunoactivation in preeclampsia: Vdelta2+ and regulatory T cells during the inflammatory stage of disease. J Reprod Immunol (2009) 80(1–2):100–8. 10.1016/j.jri.2009.01.003 19395088

[209] Jagannathan P Lutwama F Boyle MJ Nankya F Farrington LA McIntyre TI Vdelta2+ T cell response to malaria correlates with protection from infection but is attenuated with repeated exposure. Sci Rep (2017) 7(1):11487 10.1038/s41598-017-10624-3 28904345 PMC5597587

[210] Peters C Oberg HH Kabelitz D Wesch D Phenotype and regulation of immunosuppressive Vdelta2-expressing gammadelta T cells. Cell Mol Life Sci (2014) 71(10):1943–60. 10.1007/s00018-013-1467-1 24091816 PMC3997799

[211] Seidel UJ Vogt F Grosse-Hovest L Jung G Handgretinger R Lang P Gammadelta T cell-mediated antibody-dependent cellular cytotoxicity with CD19 antibodies assessed by an impedance-based label-free real-time cytotoxicity assay. Front Immunol (2014) 5:618 10.3389/fimmu.2014.00618 25520723 PMC4251440

[212] Chen Z Freedman MS. CD16+ gammadelta T cells mediate antibody dependent cellular cytotoxicity: potential mechanism in the pathogenesis of multiple sclerosis. Clin Immunol (2008) 128(2):219–27. 10.1016/j.clim.2008.03.513 18501678

[213] Tokuyama H Hagi T Mattarollo SR Morley J Wang Q So HF V gamma 9 V delta 2 T cell cytotoxicity against tumor cells is enhanced by monoclonal antibody drugs – rituximab and trastuzumab. Int J Cancer (2008) 122(11):2526–34. 10.1002/ijc.23365 18307255

[214] Gertner-Dardenne J Bonnafous C Bezombes C Capietto AH Scaglione V Ingoure S Bromohydrin pyrophosphate enhances antibody-dependent cell-mediated cytotoxicity induced by therapeutic antibodies. Blood (2009) 113(20):4875–84. 10.1182/blood-2008-08-172296 19278954

[215] Braza MS Klein B Fiol G Rossi JF Gammadelta T-cell killing of primary follicular lymphoma cells is dramatically potentiated by GA101, a type II glycoengineered anti-CD20 monoclonal antibody. Haematologica (2011) 96(3):400–7. 10.3324/haematol.2010.029520 21109686 PMC3046271

[216] Niu C Jin H Li M Xu J Xu D Hu J In vitro analysis of the proliferative capacity and cytotoxic effects of ex vivo induced natural killer cells, cytokine-induced killer cells, and gamma-delta T cells. BMC Immunol (2015) 16:61. 10.1186/s12865-015-0124-x 26458364 PMC4601131

[217] Mossner E Brunker P Moser S Puntener U Schmidt C Herter S Increasing the efficacy of CD20 antibody therapy through the engineering of a new type II anti-CD20 antibody with enhanced direct and immune effector cell-mediated B-cell cytotoxicity. Blood (2010) 115(22):4393–402. 10.1182/blood-2009-06-225979 20194898 PMC2881503

[218] Fisher JP Yan M Heuijerjans J Carter L Abolhassani A Frosch J Neuroblastoma killing properties of Vdelta2 and Vdelta2-negative gammadeltaT cells following expansion by artificial antigen-presenting cells. Clin Cancer Res (2014) 20(22):5720–32. 10.1158/1078-0432.CCR-13-3464 24893631 PMC4445920

[219] Schiller CB Braciak TA Fenn NC Seidel UJE Roskopf CC Wildenhain S CD19-specific triplebody SPM-1 engages NK and γδ T cells for rapid and efficient lysis of malignant B-lymphoid cells. Oncotarget (2016) 7(50):83392–408. 10.18632/oncotarget.13110 27825135 PMC5347777

[220] Peipp M Wesch D Oberg HH Lutz S Muskulus A van de Winkel JGJ CD20-specific immunoligands engaging NKG2D enhance gammadelta T cell-mediated lysis of lymphoma cells. Scand J Immunol (2017) 86(4):196–206. 10.1111/sji.12581 28708284

[221] Oberg HH Kellner C Gonnermann D Peipp M Peters C Sebens S Gammadelta T cell activation by bispecific antibodies. Cell Immunol (2015) 296(1):41–9. 10.1016/j.cellimm.2015.04.009 25979810

[222] Hoh A Dewerth A Vogt F Wenz J Baeuerle PA Warmann SW The activity of gammadelta T cells against paediatric liver tumour cells and spheroids in cell culture. Liver Int (2013) 33(1):127–36. 10.1111/liv.12011 23088518

[223] Zhang T Sentman CL. Cancer immunotherapy using a bispecific NK receptor fusion protein that engages both T cells and tumor cells. Cancer Res (2011) 71(6):2066–76. 10.1158/0008-5472.Can-10-3200 21282338 PMC3095211

[224] de Bruin RCG Lougheed SM van der Kruk L Stam AG Hooijberg E Roovers RC Highly specific and potently activating Vgamma9Vdelta2-T cell specific nanobodies for diagnostic and therapeutic applications. Clin Immunol (2016) 169:128–38. 10.1016/j.clim.2016.06.012 27373969

[225] de Bruin RCG Stam AGM Vangone A van Bergen en Henegouwen PMP Verheul HMW Sebestyén Z Prevention of Vγ9Vδ2 T cell activation by a Vγ9Vδ2 TCR nanobody. J Immunol (2017) 198(1):308–17. 10.4049/jimmunol.1600948 27895170

[226] Coffelt SB Kersten K Doornebal CW Weiden J Vrijland K Hau CS IL-17-producing gammadelta T cells and neutrophils conspire to promote breast cancer metastasis. Nature (2015) 522(7556):345–8. 10.1038/nature14282 25822788 PMC4475637

[227] Wakita D Sumida K Iwakura Y Nishikawa H Ohkuri T Chamoto K Tumor-infiltrating IL-17-producing gammadelta T cells support the progression of tumor by promoting angiogenesis. Eur J Immunol (2010) 40(7):1927–37. 10.1002/eji.200940157 20397212

[228] Rei M Goncalves-Sousa N Lanca T Thompson RG Mensurado S Balkwill FR Murine CD27(-) Vgamma6(+) gammadelta T cells producing IL-17A promote ovarian cancer growth via mobilization of protumor small peritoneal macrophages. Proc Natl Acad Sci U S A (2014) 111(34):E3562–70. 10.1073/pnas.1403424111 25114209 PMC4151711

[229] Caccamo N La Mendola C Orlando V Meraviglia S Todaro M Stassi G Differentiation, phenotype, and function of interleukin-17-producing human Vgamma9Vdelta2 T cells. Blood (2011) 118(1):129–38. 10.1182/blood-2011-01-331298 21505189

[230] Lo Presti E Toia F Oieni S Buccheri S Turdo A Mangiapane LR Squamous cell tumors recruit γδ T cells producing either IL17 or IFNγ depending on the tumor stage. Cancer Immunol Res (2017) 5(5):397–407. 10.1158/2326-6066.cir-16-0348 28351891

[231] Ness-Schwickerath KJ Jin C Morita CT. Cytokine requirements for the differentiation and expansion of IL-17A- and IL-22-producing human Vgamma2Vdelta2 T cells. J Immunol (2010) 184(12):7268–80. 10.4049/jimmunol.1000600 20483730 PMC2965829

[232] Voron T Colussi O Marcheteau E Pernot S Nizard M Pointet AL VEGF-A modulates expression of inhibitory checkpoints on CD8+ T cells in tumors. J Exp Med (2015) 212(2):139–48. 10.1084/jem.20140559 25601652 PMC4322048

[233] Gabrilovich DI Chen HL Girgis KR Cunningham HT Meny GM Nadaf S Production of vascular endothelial growth factor by human tumors inhibits the functional maturation of dendritic cells. Nat Med (1996) 2(10):1096–103. 10.1038/nm1096-1096 8837607

[234] Edelbauer M Datta D Vos IH Basu A Stack MP Reinders ME Effect of vascular endothelial growth factor and its receptor KDR on the transendothelial migration and local trafficking of human T cells in vitro and in vivo. Blood (2010) 116(11):1980–9. 10.1182/blood-2009-11-252460 20538805 PMC3173992

[235] Reinders ME Sho M Izawa A Wang P Mukhopadhyay D Koss KE Proinflammatory functions of vascular endothelial growth factor in alloimmunity. J Clin Invest (2003) 112(11):1655–65. 10.1172/JCI17712 14660742 PMC281640

[236] Sabatino M Kim-Schulze S Panelli MC Stroncek D Wang E Taback B Serum vascular endothelial growth factor and fibronectin predict clinical response to high-dose interleukin-2 therapy. J Clin Oncol (2009) 27(16):2645–52. 10.1200/JCO.2008.19.1106 19364969 PMC2689845

[237] Hoeres T Wilhelm M Smetak M Holzmann E Schulze-Tanzil G Birkmann J Immune cells regulate VEGF signaling via release of VEGF and antagonistic soluble VEGF receptor-1. Clin Exp Immunol (2017) 192(1):54–67. 10.1111/cei.13090 PMC5842402 29235095

[238] Glatzel A Wesch D Schiemann F Brandt E Janssen O Kabelitz D. Patterns of chemokine receptor expression on peripheral blood gamma delta T lymphocytes: strong expression of CCR5 is a selective feature of V delta 2/V gamma 9 gamma delta T cells. J Immunol (2002) 168(10):4920–9. 10.4049/jimmunol.168.10.4920 11994442

[239] Viey E Lucas C Romagne F Escudier B Chouaib S Caignard A. Chemokine receptors expression and migration potential of tumor-infiltrating and peripheral-expanded Vgamma9Vdelta2 T cells from renal cell carcinoma patients. J Immunother (2008) 31(3):313–23. 10.1097/CJI.0b013e3181609988 18317356

